# Cannabichromene: integrative modulation of apoptosis, ferroptosis, and endocannabinoid signaling in pancreatic cancer therapy

**DOI:** 10.1038/s41420-025-02674-8

**Published:** 2025-08-11

**Authors:** Yu-Na Hwang, Ju-Hee Park, Han-Heom Na, Tae-Hyung Kwon, Jin-Sung Park, Sehyun Chae, Young Taek Oh, Keun-Cheol Kim

**Affiliations:** 1 Department of Biological Sciences, College of Natural Sciences, Kangwon National University, Chuncheon, Republic of Korea; 2 Kangwon Center for System Imaging, Chuncheon, Republic of Korea; 3 Chuncheon Bioindustry Foundation, Chuncheon, Republic of Korea; 4 Korean Pharmacopuncture Institute, Seoul, Republic of Korea; 5 Division of Chemical Engineering and Bioengineering, College of Art, Culture and Engineering, Kangwon National University, Chuncheon, Republic of Korea

**Keywords:** Cell death, Chemotherapy

## Abstract

Cannabichromene (CBC: C_21_H_3_O_2_, M.W.: 314.46 g) is a non-psychotropic phytocannabinoid derived from *Cannabis sativa* (hemp), and its potential therapeutic properties have attracted increasing attention. Specifically, it has demonstrated strong anti-inflammatory effects in animal models of edema through non-CB receptor mechanisms; however, further pharmacological studies based on cancer models are required. In this study, we investigated the molecular mechanisms underlying the anti-cancer activity of CBC in human pancreatic cancer cells. Through mRNA-seq analysis, the expression levels of many genes involved in cell death pathways were upregulated or downregulated after CBC treatment, and these included ferroptosis-related genes, such as *HMOX1*. We further confirmed the functional validity of apoptosis and ferroptosis induction after CBC treatment using various molecular assays. In addition, CBC preferentially increased the expression of TRPV1 and CB2. Accordingly, the effects on cell death were reversed after treatment with TRPV1 and CB2 inhibitors, suggesting that receptor expression is necessary for the induction of apoptotic cell death. Finally, we confirmed the consistent regulation of apoptosis, ferroptosis, and endocannabinoid receptors during tumor growth inhibition after CBC treatment using in vivo xenograft models. Therefore, we propose that CBC exhibits pharmacological activity via the integrative modulation of multiple cell death pathways, which can be exploited for pancreatic cancer therapy.

## Introduction

Pancreatic cancer is one of the most lethal forms of cancer, with a 5-year patient survival rate of only 2–9% [1, 2]. Despite the limited understanding of its molecular characteristics and lack of clinically relevant pancreatic cancer subtype classifications, pancreatic ductal adenocarcinoma (PDAC) remains the most common and aggressive form, with an extremely poor prognosis [3]. PDAC is often diagnosed at advanced stages and is resistant to most treatment modalities, making it a key focus of cancer research regarding novel mechanisms to achieve effective treatment [4].

Cannabinoids extracted from *Cannabis sativa* exert their effects by binding to specific receptors that play a role in tissue development and homeostasis maintenance in the human body [5]. More than 150 cannabinoids have been identified, including cannabinol and Δ^9^-tetrahydrocannabinol (Δ^9^-THC), cannabidiol (CBD), cannabigerol, and cannabichromene (CBC) [6]. Among these, Δ^9^-THC and CBD have received the most attention regarding their medical applications [7], and both have pharmacological effects, including pain relief, suppressing anxiety, and potential therapeutic effects on neurodegenerative diseases and cancers [8]. Moreover, cannabinoids can enhance the effectiveness of conventional chemotherapy and radiotherapy when used in combination, while alleviating side effects, making them promising candidates for integrated cancer treatment strategies [9]. Non-psychoactive CBD has gained increasing attention within the scientific community owing to its potential pharmacological use based on various disease models, unlike Δ^9^-THC, which has psychotropic effects [10]. Specifically, CBD inhibits the malignant growth of several solid tumors, including gliomas [11], breast cancer [12], and prostate cancer [13]. Its anti-cancer activity is multifaceted and involves cell cycle arrest, the inhibition of proliferation, the induction of autophagy and apoptosis, and the suppression of cell adhesion, migration, angiogenesis, and metastasis [14, 15].

CBC has diverse therapeutic benefits, including anti-inflammatory [16], anticonvulsant [17], antibacterial [18], and antinociceptive effects [19]. It also reduces the production of nitrite, INF-γ, and IL-10 in lipopolysaccharide (LPS)-stimulated peritoneal macrophages. Its anti-inflammatory effects have been further demonstrated using animal models, where CBC reduces inflammation and LPS-induced edema in the paws of mice, with a synergistic effect observed when co-administered with Δ^9^-THC [20]. The anticonvulsant potential of CBC has been highlighted using *Scn1a*^+/−^ mouse models of Dravet syndrome and zebrafish models, with CBC being as effective as CBD at reducing seizures [21]. In addition, CBC has antinociceptive properties in tail-flick assays, formalin-induced inflammatory pain models, and cisplatin-induced peripheral neuropathy models, indicating a broad range of pain-relieving applications [19]. It also synergistically interacts with Δ^9^-THC to promote urothelial carcinoma cell death and inhibit migration [22]. Given the increasing interest in cannabinoid use for medicinal purposes, research on minor cannabinoids, such as CBC, is increasing. In this study, we investigated the potential mechanisms underlying the effects of CBC on pancreatic cancer, focusing on the integrative regulation of cell death pathways, including apoptosis and ferroptosis, and the activation of endocannabinoid receptors.

## Results

### CBC treatment induces apoptotic cell death in pancreatic cancer cells

**Fig. 1:**
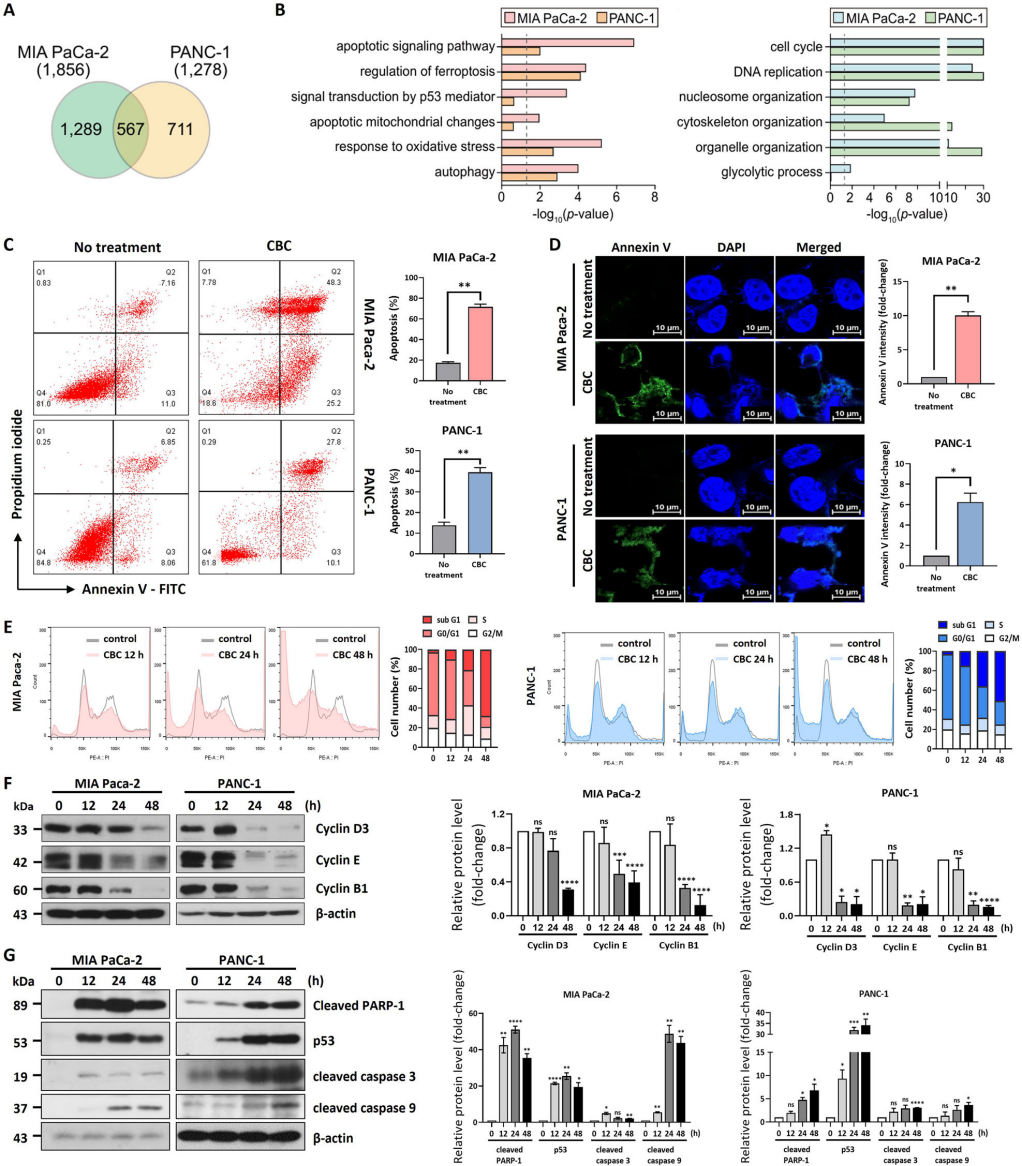
Cannabichromene (CBC) treatment induces cell death via the apoptotic pathway in human pancreatic cancer cells. **A** mRNA-seq analysis identified 2567 differentially expressed genes (DEGs) in two MIA PaCa-2 and PANC-1 pancreatic cancer cells. **B** Gene Ontology (GO) and KEGG pathway analyses showed the relative enrichment of the apoptotic signaling pathway, regulation of ferroptosis, cell cycle, and DNA replication. Statistical significance is displayed as the −log_10_ (*p*-value), where the enrichment *p*-value was obtained using DAVID software. The dotted line indicates the *p*-value cutoff used. Left: upregulated genes; right: downregulated genes. **C** Annexin V-FITC/propidium iodide (PI) staining analysis was performed using pancreatic cancer cells after CBC treatment for 24 h. The graph represents the number of increased apoptotic MIA PaCa-2 and PANC-1 cells, respectively. Statistical significance was determined using Student’s *t* test, with *P* ≤ 0.05. **D** An annexin V-FITC fluorescence image was also captured from the cultured pancreatic cancer cells based on the same treatment condition. **E** FACS analysis was performed on CBC-treated pancreatic cancer cells. CBC treatment resulted in an increased dead cell population after 24 h. Statistical significance was determined using Student’s *t* test, with *P* ≤ 0.05. **F** Western blot analysis also performed on cyclin proteins in CBC-treated pancreatic cancer cells. Expression levels of most cyclin proteins were downregulated after CBC treatment. The graphs were obtained based on densitometric data from western blot images. **G** Western blot analysis was performed on two pancreatic cancer cells after CBC treatment. The expression of cleaved PARP-1, p53, and cleaved caspase-3 and 9 was increased in CBC-treated cancer cells. The graphs were obtained based on densitometric data from western blot images. Statistical significance was determined using Student’s *t* tests, with *P* ≤ 0.05 indicating statistical significance. Results are expressed as mean ± SD (*n* = 3). Statistical analysis was performed with one-way ANOVA followed by Dunnett’s multiple comparison test, with *P* < 0.01 or *P* < 0.001 vs. 0 h. MIA PaCa-2 and PANC-1 cells were treated with 35 μM and 30 μM CBC, respectively.

First, we performed an MTT assay to determine the IC_50_ of CBC using the two pancreatic cancer cell lines. The estimated IC_50_s of CBC were ~35 µM for MIA PaCa-2 cells and 30 µM for PANC-1 cells (Supplementary Fig. S1), indicating that these concentrations could be used in subsequent experiments. To identify genes related to the pharmacological activity of CBC, we performed mRNA sequencing 24 h after CBC treatment using both pancreatic cancer cell lines (Fig. 1A). Here, 2567 genes were differentially expressed between the pancreatic cancer cell lines. Among these, 1856 DEGs were identified in MIA PaCa-2 cells and 1278 DEGs were identified in PANC-1 cells. The upregulated genes were significantly associated with cell death processes, including apoptosis, the p53 signaling pathway, ferroptosis, and autophagy, whereas the downregulated genes were mainly related to cell proliferation processes, such as the cell cycle, DNA replication, cytoskeleton organization, and glycolysis (Fig. 1B). Annexin V-FITC/PI staining was performed to quantify the dead cell population in CBC-treated pancreatic cancer cells (Fig. 1C). Annexin V-FITC-positive cells increased to 73.5% in CBC-treated MIA PaCa-2 cells compared to 18.16% in the control group and to 37.9% in CBC-treated PANC-1 cells compared to 14.91% in the control group. CBC treatment increased both early and late apoptotic cell populations in pancreatic cancer cells. Consistently, fluorescence images showed increased Annexin V-FITC staining in CBC-treated pancreatic cancer cells, indicating that CBC induced apoptotic cell death (Fig. 1D). FACS analysis was performed to examine the cell cycle distribution following CBC treatment (Fig. 1E), revealing an increased dead cell population after 24 h, suggesting that CBC induces cell death by promoting cell cycle exit. We also performed western blot analysis of cell cycle-related proteins to confirm the cell cycle alterations caused by CBC treatment (Fig. 1F). Expression levels of most cyclins, including cyclin D3, E, and B1, were downregulated after CBC treatment, indicating that CBC promotes cell cycle exit by decreasing cyclin expression. Next, we investigated apoptotic cell death-related proteins using western blotting following CBC treatment in pancreatic cancer cells (Fig. 1G). This increased expression of the tumor suppressor p53 and elevated the levels of cleaved forms of PARP-1, caspase-3, and caspase-9. These data suggest that CBC induces apoptosis in pancreatic cancer cells.

### CBC induces ferroptotic cell death via ferroptosis-related gene activation

**Fig. 2:**
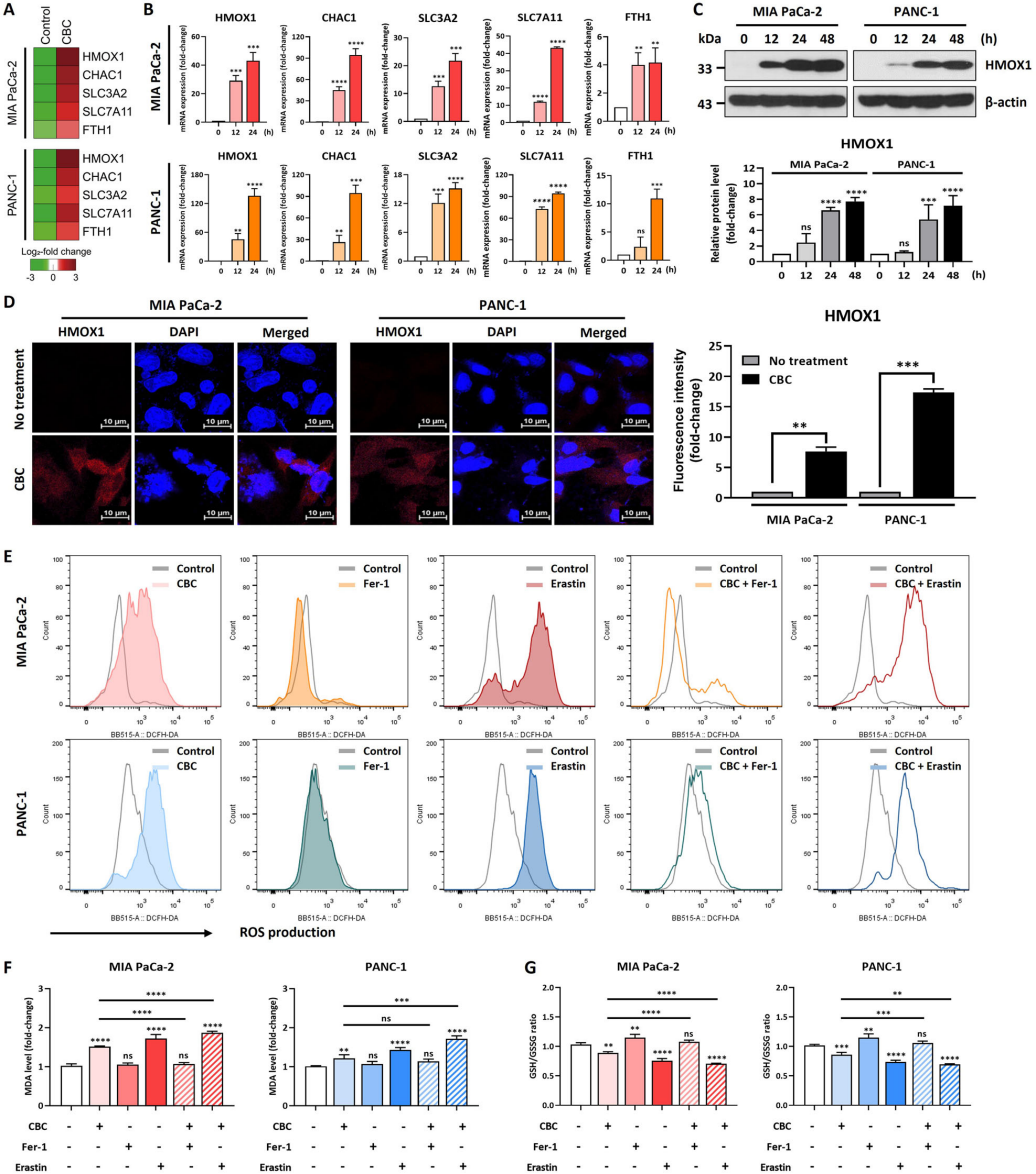
Cannabichromene (CBC) treatment induces cell death via a ferroptosis-related signaling pathway. **A** Heatmap of ferroptosis-related genes, obtained from mRNA-seq data. **B** Quantitative PCR analysis of ferroptosis-related genes, performed using CBC-treated pancreatic cancer cells. *HMOX1*, *CHAC1*, *SLC3A2*, *SLC7A11*, and *FTH1* levels were quickly increased by CBC treatment at the transcriptional level. **C** Western blot analysis showed that HMOX1 protein expression was upregulated by CBC treatment. The graphs were obtained from densitometric data for western blot images. Results are expressed as mean ± SD (*n* = 3). Statistical analysis was performed with one-way ANOVA followed by Dunnett’s multiple comparison test, with *P* < 0.01 or *P* < 0.001 vs. 0 h. **D** Increased HMOX1 protein expression examined by performing an immunostaining experiment. Statistical significance was determined using Student’s *t* test, with *P* ≤ 0.05. **E** We performed flow cytometric analysis using H_2_DCFDA to measure the reactive oxygen species (ROS) level after CBC treatment. The ROS level was increased in CBC-treated pancreatic cancer cells but slightly restored after combined treatment with CBC and ferrostatin, a ferroptosis inhibitor. However, the ROS level was severely increased after combination treatment with CBC and erastin, a ferroptosis inducer. **F** Lipid peroxidation in pancreatic cancer cells was examined after CBC treatment. The malondialdehyde (MDA) level was increased in CBC-treated cancer cells but downregulated after combined treatment with CBC and ferrostatin. However, the MDA level was severely increased after combination treatment with CBC and erastin. Results are expressed as mean ± SD (*n* = 3). Statistical analysis was performed with one-way ANOVA followed by Dunnett’s multiple comparison test, with *P* < 0.01 or *P* < 0.001 vs. no treatment. **G** The GSH/GSSG ratio was examined using a GSH kit. All data are similar to the data obtained from the ROS and lipid peroxidation analysis. Statistical significance was determined using Student’s *t* tests, with *p*-values ≤ 0.05 indicating statistical significance. Results are expressed as mean ± SD (*n* = 3). Statistical analysis was performed with one-way ANOVA followed by Dunnett’s multiple comparison test, with *P* < 0.01 or *P* < 0.001 vs. no treatment. MIA PaCa-2 and PANC-1 cells were treated with 35 μM and 30 μM CBC, respectively. Additional treatments included 10 μM ferrostatin-1 (Fer-1) and erastin.

Transcriptome analysis revealed that CBC significantly upregulated the expression of genes related to ferroptosis and those involved in apoptosis (Fig. 1B). Therefore, we determined whether ferroptosis pathways were activated in CBC-treated pancreatic cancer cells. Heatmap data indicated that CBC treatment upregulated the expression of ferroptosis-related genes, including *HMOX1* (Fig. 2A). We further performed qRT-PCR on five selected ferroptosis-related genes (*HMOX1*, *CHAC1*, *SLC3A2*, *SLC7A11*, and *FTH1*) in pancreatic cancer cells after 12 and 24 h of CBC treatment (Fig. 2B). CBC treatment significantly increased the expression of these genes in a time-dependent manner. Consistently, HMOX1 protein levels were significantly elevated following CBC treatment, as shown through western blot analysis, emphasizing that it acts as a key regulator of CBC ferroptosis in pancreatic cancer cells (Fig. 2C). The upregulation of HMOX1 expression was further confirmed based on immunofluorescence staining, as HMOX1 localization was observed in both the nucleus and cytoplasm (Fig. 2D). Additionally, we measured cellular ROS levels after CBC treatment using H_2_DCFDA and flow cytometry (Fig. 2E). These were increased by treatment with either CBC or erastin (a ferroptosis inducer); however, no significant increase was observed after ferrostatin (a ferroptosis inhibitor) treatment. However, the elevated ROS levels induced by CBC were slightly reduced when CBC was combined with ferrostatin, whereas ROS levels were additively increased when CBC was combined with erastin. To further investigate lipid peroxidation, an essential process in ferroptosis, we measured malondialdehyde (MDA) levels (Fig. 2F). These were elevated following treatment with either CBC or erastin. However, the CBC-induced increase in MDA levels was reversed after co-treatment with ferrostatin, suggesting that lipid peroxidation caused by CBC is closely regulated by ferroptosis inhibition. We also measured GSH levels to examine the redox imbalance associated with ferroptosis (Fig. 2G). Similar to that with erastin, CBC reduced the GSH/GSSG ratio, whereas ferrostatin increased it. Notably, the decrease in the GSH/GSSG ratio induced by CBC was restored after co-treatment with ferrostatin, indicating that the redox imbalance induced by CBC is tightly regulated by ferroptosis inhibition. In conclusion, CBC induces ferroptotic cell death by increasing ROS production and lipid peroxidation through the activation of ferroptosis-related genes, including *HMOX1*.

### Ferroptosis inhibition delays CBC-induced cell death

**Fig. 3:**
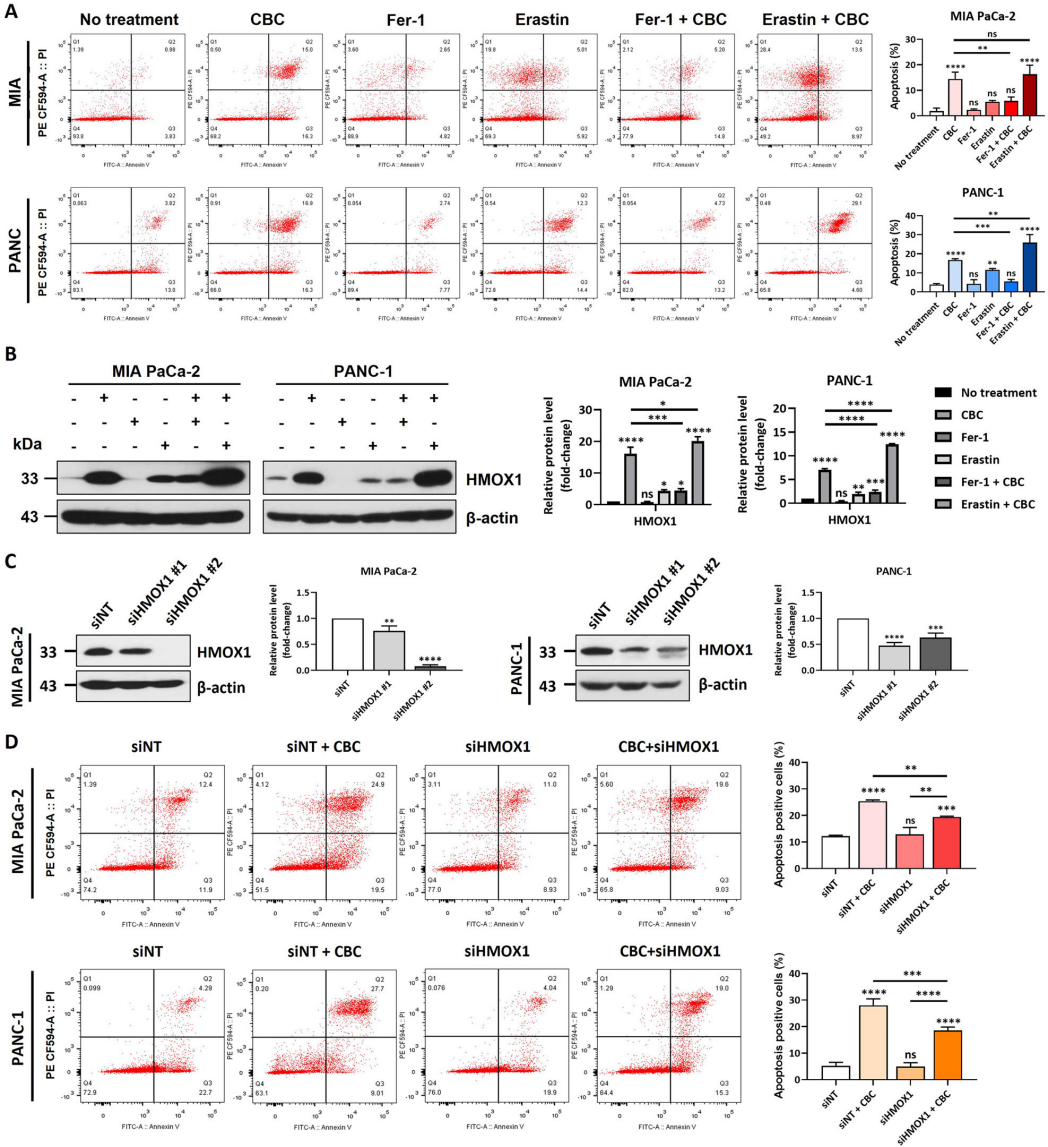
Inhibition on ferroptosis delays cannabichromene (CBC)-induced cell death. **A** Annexin V-FITC/propidium iodide (PI) staining analysis confirmed that a ferrostatin-1 and erastin 1 h pre-treatment affected CBC-induced cell death. Results are expressed as mean ± SD (*n* = 3). Statistical analysis was performed with one-way ANOVA followed by Dunnett’s multiple comparison test, with *P* < 0.01 or *P* < 0.001 vs. no treatment. **B** Western blot analysis showing the change in HMOX1 protein expression induced by ferrostatin-1, erastin, and CBC treatment. Results are expressed as mean ± SD (*n* = 3). Statistical analysis was performed with one-way ANOVA followed by Dunnett’s multiple comparison test, with *P* < 0.01 or *P* < 0.001 vs. no treatment. **C** Two siRNAs for HMOX1 were prepared and transfected into both MIA PaCa-2 and PANC-1 pancreatic cancer cells. The western blot image shows that HMOX1 expression was successfully downregulated after siRNA transfection in the two pancreatic cancer cell lines. Results are expressed as mean ± SD (*n* = 3). Statistical analysis was performed with one-way ANOVA followed by Dunnett’s multiple comparison test, with *P* < 0.01 or *P* < 0.001 vs. siNT. **D** Annexin V-FITC/PI staining analysis showed that HMOX1 inhibition affected CBC-induced cell death. Statistical significance was determined using Student’s *t* tests, with *p*-values ≤ 0.05 indicating statistical significance. Results are expressed as mean ± SD (*n* = 3). Statistical analysis was performed with one-way ANOVA followed by Dunnett’s and Tukey’s multiple comparison test, with *P* < 0.01 or *P* < 0.001 vs. siNT. MIA PaCa-2 and PANC-1 cells were treated 35 μM and 30 μM CBC, respectively. Additional treatments included 10 μM Fer-1 and erastin.

Annexin V/PI analysis was performed to investigate whether ferrostatin (a ferroptosis inhibitor) or erastin (a ferroptosis activator) can regulate CBC-induced cell death (Fig. 3A). As expected, ferrostatin significantly reduced CBC-induced cell death, whereas erastin had no notable effect. Additionally, western blot analysis revealed that ferrostatin and CBC co-treatment drastically decreased HMOX1 expression, suggesting that the regulatory effect of ferrostatin on ferroptosis might be mediated by HMOX1 (Fig. 3B). To confirm the role of HMOX1 in CBC-induced cell death, we used siRNA knockdown. Western blot analysis confirmed that HMOX1 protein levels were successfully reduced after siHMOX1 transfection in both cancer cell lines (Fig. 3C). Annexin V/PI staining showed that the dead cell population induced by CBC treatment was significantly decreased following HMOX1 knockdown (Fig. 3D). These findings suggest that inhibition of the ferroptotic pathway delays CBC-induced cell death, highlighting the crucial role of HMOX1 in mediating this process.

### Combination treatment with CBC and cell death inhibitors restores cell viability

**Fig. 4:**
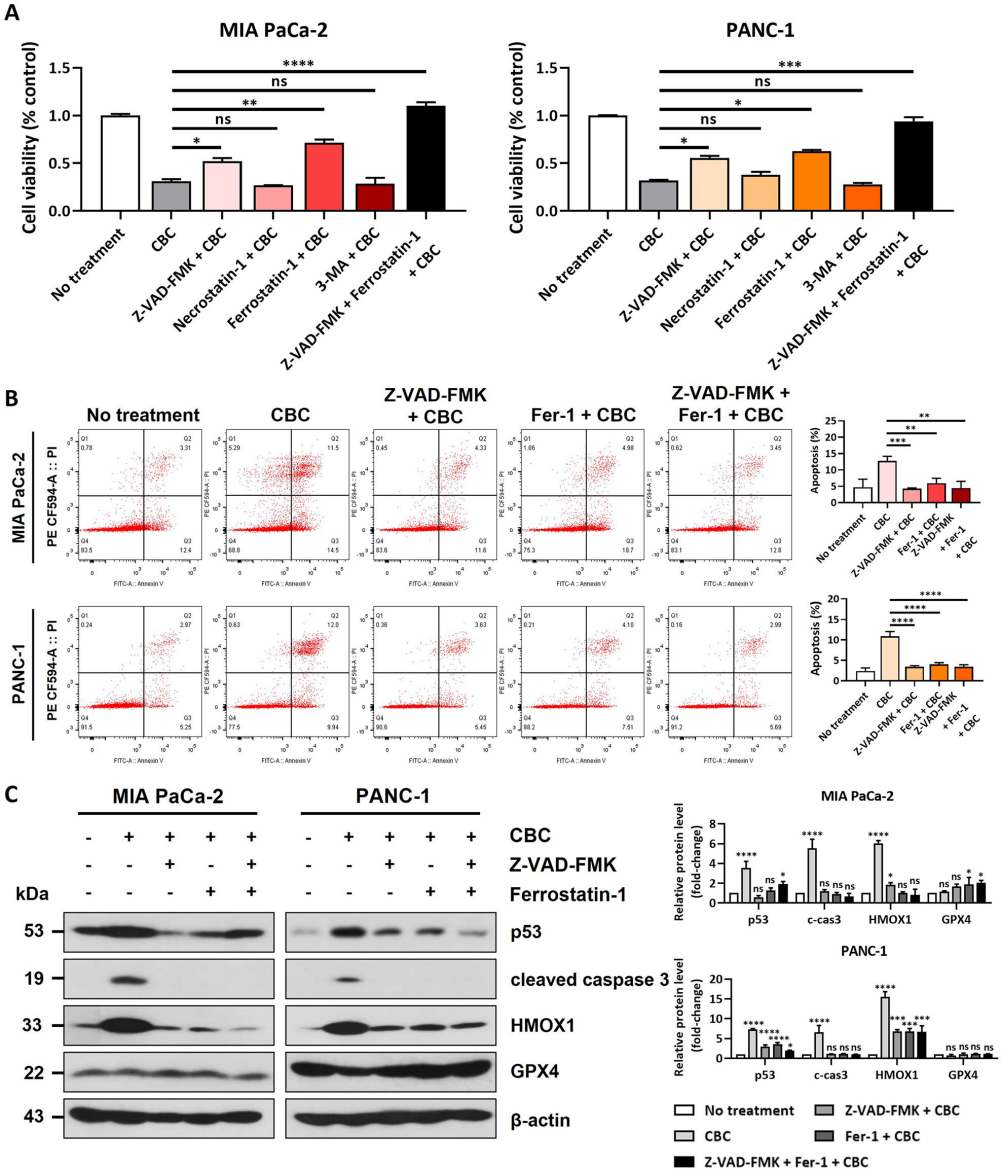
Combined inhibition of apoptosis and ferroptosis delays cannabichromene (CBC)-induced cell death. **A** An MTT assay was performed to confirm cell viability in both pancreatic cancer cell lines using several inhibitors related to cell death-related pathways. Treatment with Z-VAD-FMK or ferrostatin slightly restored the anti-proliferative activity of CBC in human pancreatic cells. **B** Annexin V-FITC/propidium iodide (PI) staining analysis showed that the dual inhibitory effects on apoptosis and ferroptosis inhibition additionally restored the CBC-induced dead cell population. Results are expressed as mean ± SD (*n* = 3). Statistical analysis was performed with one-way ANOVA followed by Tukey’s multiple comparison test, with *P* < 0.01 or *P* < 0.001 vs. no treatment or CBC. **C** Western blot analysis confirmed that protein expression was changed by CBC, Z-VAD-FMK, and ferrostatin-1. Statistical significance was determined using Student’s *t* tests, with *p*-values ≤ 0.05 indicating statistical significance. Results are expressed as mean ± SD (*n* = 3). Statistical analysis was performed with one-way ANOVA followed by Dunnett’s multiple comparison test, with *P* < 0.01 or *P* < 0.001 vs. no treatment. MIA PaCa-2 and PANC-1 cells were treated with 35 μM and 30 μM CBC, respectively. Additional treatments included 10 μM Fer-1, erastin, and necrostatin-1, as well as 20 μM Z-VAD-FMK.

To clarify the regulatory effect of CBC on both apoptotic and ferroptotic cell death, we investigated the effects of combined treatment with CBC and several inhibitors, including Z-VAD-FMK, necrostatin-1, ferrostatin-1, and 3-MA. MTT assays revealed that cell viability was decreased after CBC treatment but restored after pretreatment with Z-VAD-FMK (an apoptosis inhibitor) and ferrostatin-1 (a ferroptosis inhibitor) (Fig. 4A). However, we did not observe the significant restoration of cell viability following combined treatment with necrostatin-1 (a necroptosis inhibitor) or 3-MA (an autophagy inhibitor). Interestingly, this restoration of cell viability was not detected when CBC was combined with Δ^9^-THC or CBD treatment (Supplementary Fig. S2). Notably, cell viability was dramatically improved after simultaneous pretreatment with Z-VAD-FMK and ferrostatin-1, suggesting that CBC specifically regulates both apoptotic and ferroptotic signaling pathways. Consistent results were obtained from the Annexin V/PI staining experiment, which showed the restoration of cell viability after combined treatment with CBC and the inhibitors (Fig. 4B). Western blot analysis further confirmed that cell death-related proteins were tightly regulated upon combined treatment with CBC with apoptosis and ferroptosis inhibitors (Fig. 4C). These results suggest that CBC induces cell death in pancreatic cancer cells through both apoptotic and ferroptotic signaling pathways and that this can be modulated by combined treatment with specific inhibitors.

### CBC treatment regulates TRPV1 and CB_2_ receptor expression

**Fig. 5:**
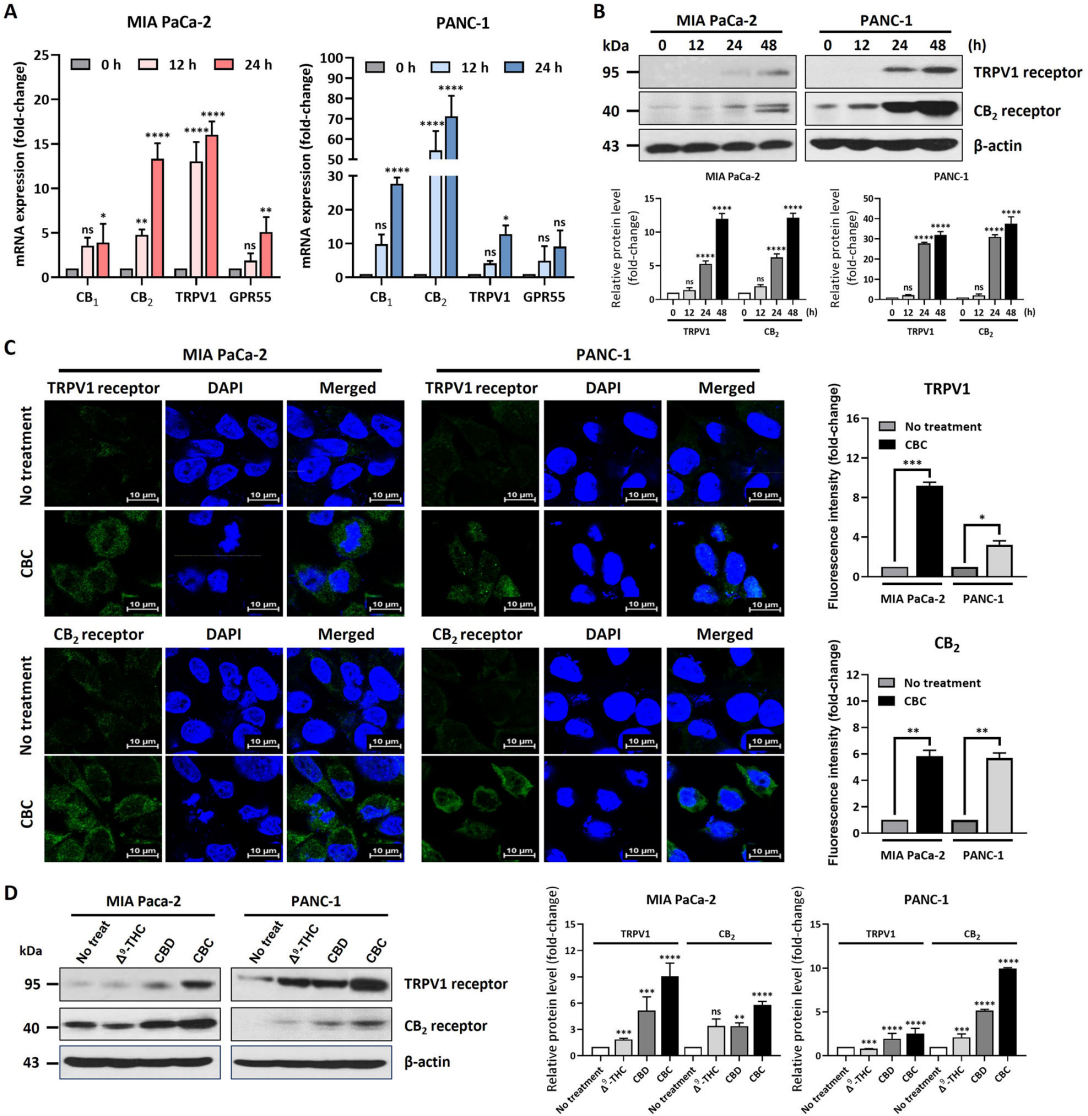
Cannabichromene (CBC) treatment increases the expression of CB_2_ and TRPV1 receptors in pancreatic cancer cells. **A** qRT-PCR analysis was performed to examine the expression of endocannabinoid receptors in CBC-treated pancreatic cancer cells. The expression of CB_2_ and TRPV1 receptor-encoding genes was increased by CBC treatment. Results are expressed as mean ± SD (*n* = 3). Statistical analysis was performed with one-way ANOVA followed by Dunnett’s multiple comparison test, with *P* < 0.01 or *P* < 0.001 vs. CTRL. **B** Western blot analysis of CB_2_ and TRPV1 receptors was performed on CBC-treated pancreatic cancer cells. The expression of CB_2_ and TRPV1 receptors was increased in CBC-treated cancer cells. The graphs were obtained from densitometric data based on western blot images. Results are expressed as mean ± SD (*n* = 3). Statistical analysis was performed with one-way ANOVA followed by Dunnett’s multiple comparison test, with *P* < 0.01 or *P* < 0.001 vs. CTRL. **C** Immunofluorescence analysis was also performed using CBC-treated pancreatic cancer cells. The expression of CB_2_ and TRPV1 receptor was increased by CBC treatment. The graphs were obtained from fluorescence images. **D** We also compared the expression of CB_2_ and TRPV1 receptors after treatment with three cannabinoids (tetrahydrocannabinol [THC], cannabidiol [CBD], and CBC). The three cannabinoids increased the expression of CB_2_ and TRPV1 receptors, but the effects of CBC were highly sensitive to the expression of the two receptors. The graphs were obtained from densitometric data based on western blot images. Statistical significance was determined using Student’s *t* tests, with *p*-values ≤ 0.05 indicating statistical significance. Results are expressed as mean ± SD (*n* = 3). Statistical analysis was performed with one-way ANOVA followed by Dunnett’s multiple comparison test, with *P* < 0.01 or *P* < 0.001 vs. CTRL. MIA PaCa-2 and PANC-1 cells were treated with 35 μM and 30 μM CBC, respectively. Additional treatments included 50 μM Δ^9^-THC and 20 μM CBD.

Previous studies have suggested that CBD and ∆^9^-THC exert anti-cancer effects through endocannabinoid system (ECS)-related receptors in several types of cancer, including gliomas [23]. To investigate the relevance of this with regard to CBC, we examined the expression of several receptors after CBC treatment via qRT-PCR (Fig. 5A). Levels of most receptors responded to CBC treatment; however, the expression of *TRPV1* and *CB2* receptors was notably increased at the mRNA level. Western blot and immunostaining analyses showed that TRPV1 and CB2 receptor expression was elevated near the cytoplasm and cell membrane after CBC treatment in a time-dependent manner (Fig. 5B, C). Additionally, we compared the regulatory effects of various cannabinoids, including CBC, CBD, and ∆^9^-THC, on TRPV1 and CB2 receptors (Fig. 5D). Western blot analysis revealed that CBC treatment resulted in the more pronounced regulation of CB2 and TRPV1 expression, compared to that with THC and CBD. These findings suggest that CBC induces cell death by upregulating TRPV1 and CB2 expression, acting more specifically than the other cannabinoids.

### Downregulation of TRPV1 and CB_2_ expression rescues CBC-induced apoptotic cell death

**Fig. 6:**
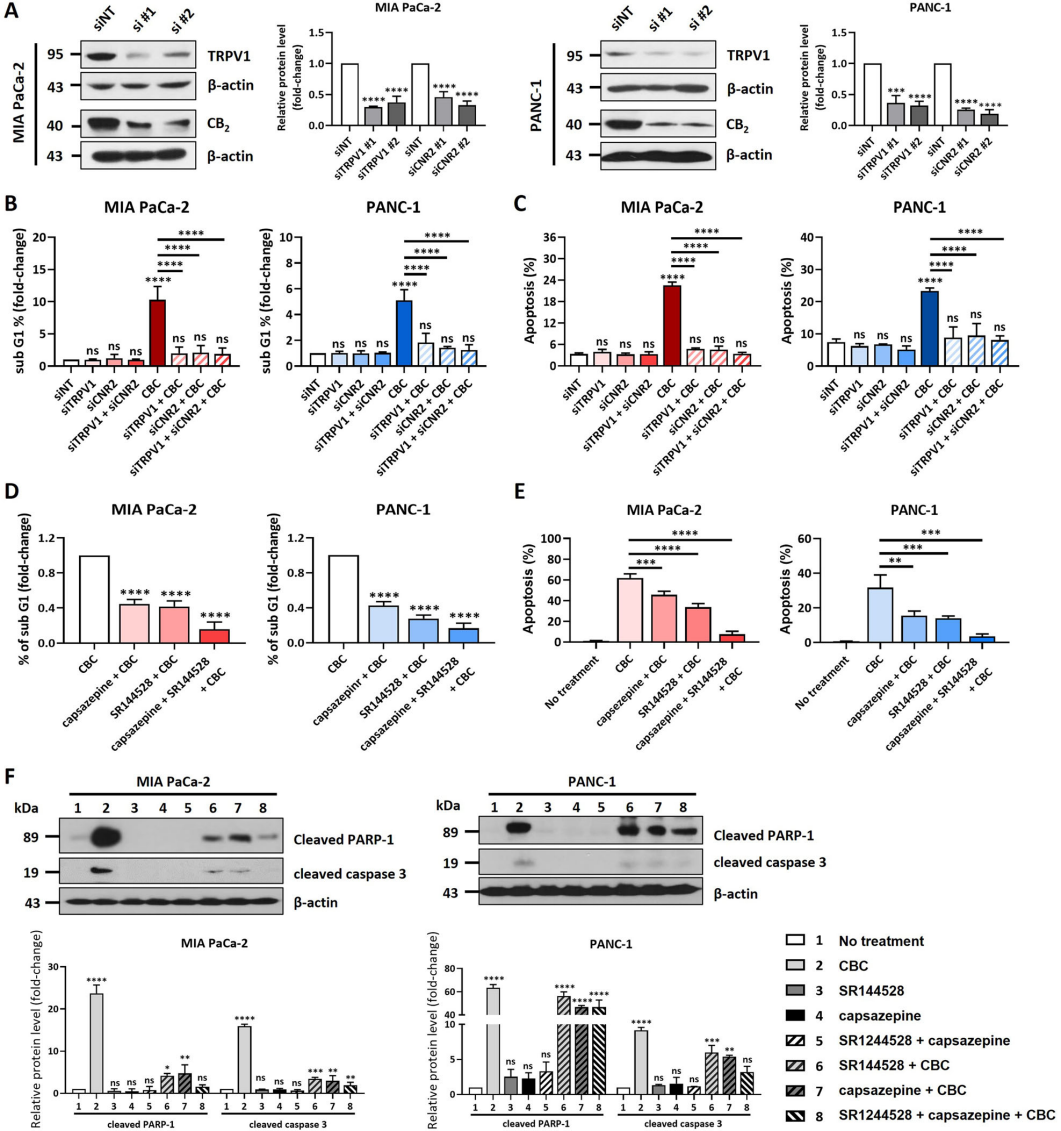
Inhibition of CB_2_ and TRPV1 receptors blocks cannabichromene (CBC)-induced apoptotic cell death. **A** Two siRNAs each targeting CB_2_ and TRPV1 were prepared and transfected into both MIA PaCa-2 and PANC-1 pancreatic cancer cells. The western blot image shows that CB_2_ and TRPV1 levels were successfully downregulated after siRNA transfection in the two pancreatic cancer cells. Results are expressed as mean ± SD (*n* = 3). Statistical analysis was performed with one-way ANOVA followed by Dunnett’s multiple comparison test, with *P* < 0.01 or *P* < 0.001 vs. CTRL. **B** The graphs were prepared after performing FACS analysis. CBC treatment increased the dead cell population in both pancreatic cancer cell lines, but this was severely decreased after siCB_2_ and siTRPV1 transfections. Results are expressed as mean ± SD (*n* = 3). Statistical analysis was performed with one-way ANOVA followed by Tukey’s multiple comparison test, with *P* < 0.01 or *P* < 0.001 vs. CTRL or CBC. **C** The graphs were prepared based on Annexin V-FITC/propidium iodide (PI) staining analysis. We also obtained similar data for the Annexin V-FITC/PI staining analysis. Results are expressed as mean ± SD (*n* = 3). Statistical analysis was performed with one-way ANOVA followed by Tukey’s multiple comparison test, with *P* < 0.01 or *P* < 0.001 vs. CTRL or CBC. **D** The graphs were prepared based on FACS analysis after treatment with cannabinoid inhibitors. The increased in the dead cell population induced by CBC treatment was dramatically decreased by both capsazepine and SR144528. Results are expressed as mean ± SD (*n* = 3). Statistical analysis was performed with one-way ANOVA followed by Dunnett’s multiple comparison test, with *P* < 0.01 or *P* < 0.001 vs. CBC. **E** The graphs were prepared based on Annexin V-FITC/PI staining analysis after treatment with cannabinoid inhibitors. We also obtained similar data for the Annexin V-FITC/PI staining analysis. Results are expressed as mean ± SD (*n* = 3). Statistical analysis was performed with one-way ANOVA followed by Tukey’s multiple comparison test, with *P* < 0.01 or *P* < 0.001 vs. CBC. **F** Western blot analysis was performed to examine whether apoptotic cell death is influenced after inhibition of the CB_2_ and TRPV1 receptor. CBC treatment increased cleaved forms of PARP-1 and caspase-3, but this was decreased after combination treatment with CBC and cannabinoid receptor inhibitors. The graphs were obtained using densitometric data from western blot images. Statistical significance was determined using Student’s *t* tests, with *p*-values ≤ 0.05 indicating statistical significance. Results are expressed as mean ± SD (*n* = 3). Statistical analysis was performed with one-way ANOVA followed by Dunnett’s multiple comparison test, with *P* < 0.01 or *P* < 0.001 vs. CTRL. MIA PaCa-2 and PANC-1 cells were treated with 35 μM and 30 μM CBC, respectively. Additional treatments included 5 μM capsazepine and 10 μM SR144528.

CBC treatment not only induced apoptosis and ferroptosis but also increased the expression of CB2 and TRPV1 receptors. To explore the potential interactions between ECS receptors and cell death, we performed inhibition experiments targeting CB2 and TRPV1. First, we prepared siRNAs targeting CB2 and TRPV1, transfected them into two pancreatic cancer cell lines, and confirmed knockdown via western blot analysis (Fig. 6A). FACS analysis revealed that the increase in the sub-G1 phase caused by CBC treatment was reversed after CB2 and TRPV1 knockdown (Fig. 6B; Supplementary Fig. S3A). Consistently, Annexin V/PI staining showed that the siRNA-mediated knockdown of these receptors rescued the apoptosis-positive cell population induced by CBC (Fig. 6C; Supplementary Fig. S3B). To further validate these results, pancreatic cancer cells were pretreated with capsazepine, a TRPV1 receptor antagonist, and SR144528, a CB2 receptor antagonist, prior to CBC treatment. These inhibitors exerted similar effects as the siRNA, indicating that blocking CB2 and TRPV1 affected CBC-induced cell death (Fig. 6D, E; Supplementary Fig. S4A, B). Next, we investigated the involvement of apoptosis in ECS expression by examining the cleaved forms of PARP-1 and caspase-3, two typical apoptotic markers (Fig. 6F). Western blot analysis demonstrated that the CBC-induced increase in cleaved PARP-1 and caspase-3 levels was reversed by receptor inhibition. In addition, we examined the relationship between ferroptosis and ECS receptor expression based on HMOX1 levels. Although HMOX1 expression was upregulated by CBC treatment, its expression was not affected by receptor inhibition (Supplementary Fig. S5). Thus, we suggest that TRPV1 and CB2 inhibition rescues CBC-induced apoptotic cell death, but not ferroptosis.

### CBC treatment inhibits pancreatic tumor growth in a xenograft mouse model

**Fig. 7:**
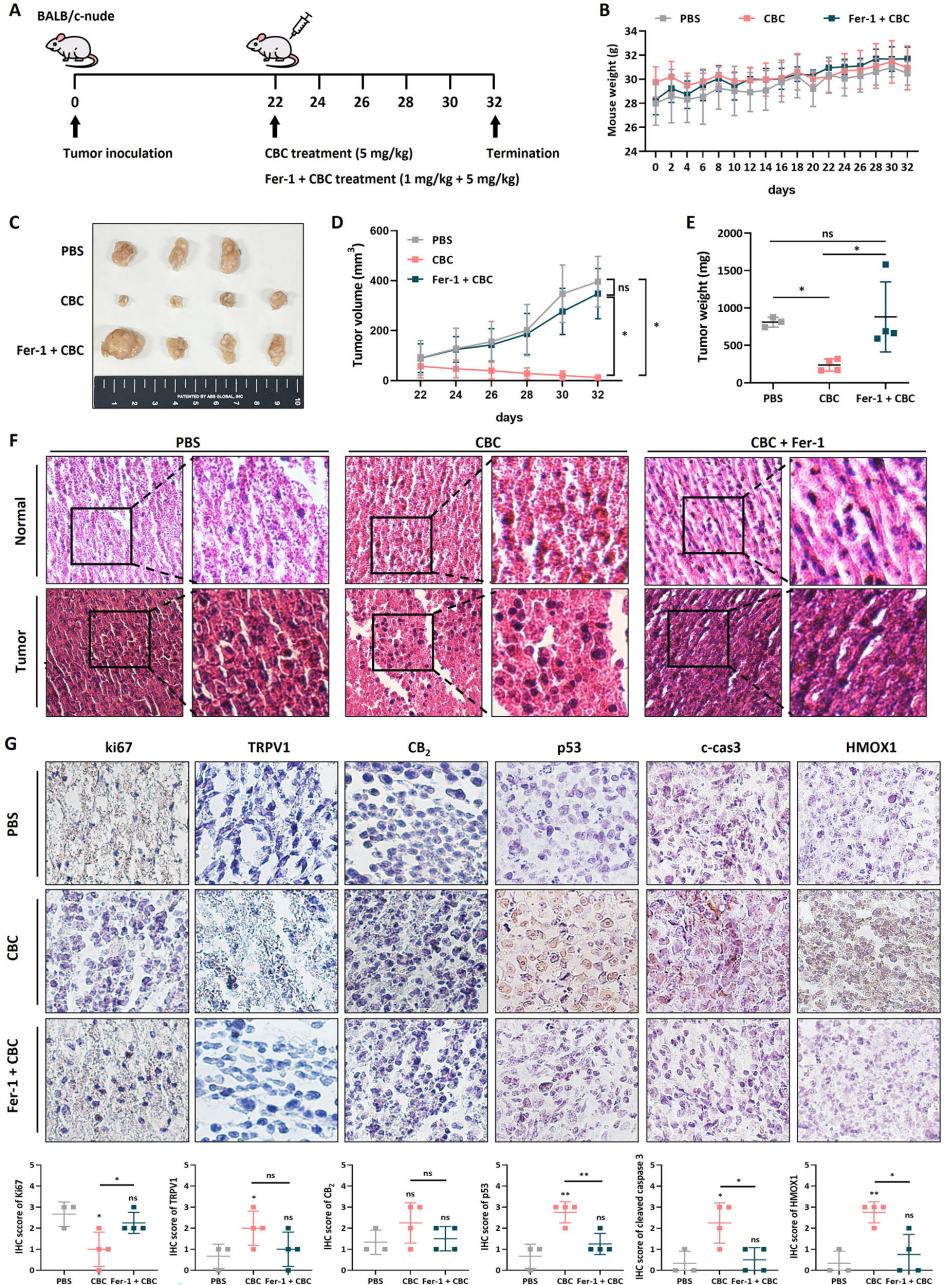
Cannabichromene (CBC) treatment results in tumor regression in mouse xenograft models. **A** A schematic diagram is presented showing the timeline of the tumor xenograft model using BALB/c-nude mice and CBC treatment. **B** Mouse weights were measured every other day after tumor inoculation. **C** Tumor size, **D** tumor volume, and **E** tumor weight were measured after euthanizing the mice. CBC resulted in a decrease in the tumor size and volume, but these effects on the size and volume were not observed with combination treatment comprising CBC and ferrostatin. Results are expressed as mean ± SD (PBS (*n* = 3), CBC (*n* = 4), Fer-1 + CBC (*n* = 4)). Statistical analysis was performed with one-way ANOVA followed by Tukey’s multiple comparison test, with *P* < 0.01 or *P* < 0.001 vs. PBS or CBC. **F** Representative H&E staining images were compared between normal and tumor regions. **G** Immunohistochemistry (IHC) images were obtained for apoptotic marker proteins, including p53, cleaved PARP-1, and cleaved caspase-3. IHC images were obtained for ferroptosis marker proteins, including HMOX1 and SLC7A11. IHC images were also obtained for endocannabinoid-related proteins, including CB_2_ and TRPV1 receptor proteins. The graphs were generated based on densitometric data from IHC images. Statistical significance was determined using Student’s *t* tests, with *p*-values ≤ 0.05 indicating statistical significance. Results are expressed as mean ± SD (PBS (*n* = 3), CBC (*n* = 4), Fer-1 + CBC(*n* = 4)). Statistical analysis was performed with one-way ANOVA followed by Tukey’s multiple comparison test, with *P* < 0.01 or *P* < 0.001 vs. PBS or CBC.

Our novel findings suggest that CBC treatment induces HMOX1-dependent ferroptosis in pancreatic cancer cells. To confirm these findings in vivo, we developed a xenograft model using nude mice over 3 weeks. Mice were treated with CBC or a combination of ferrostatin and CBC every 2 days via direct injection into the tumor (Fig. 7A). No significant weight loss was observed during CBC or ferrostatin treatment, indicating that CBC did not cause severe side effects in the mice (Fig. 7B). However, tumor mass, volume, and weight were significantly reduced by CBC treatment (Fig. 7C–E). No tumor regression was observed with the combined CBC and ferrostatin treatment, suggesting that CBC-mediated tumor regression is dependent on ferroptosis in pancreatic tumors. Histological analysis via H&E staining showed more significant cell death in tumors from mice treated with CBC compared to that in the PBS-treated or combination treatment groups (Fig. 7F). These results indicated that CBC treatment inhibits the growth of pancreatic tumors via ferroptosis in a xenograft mouse model. Tumor tissues were collected from euthanized mice and subjected to IHC staining for cell death markers and cannabinoid receptors (Fig. 7G). Ki67 expression was downregulated in CBC-treated mice compared to that in the PBS or combination treatment groups. In contrast, apoptosis- and ferroptosis-associated marker proteins were increased in tumors from CBC-treated mice. Additionally, endocannabinoid receptor expression was elevated in CBC-treated mice. Thus, CBC appears to regulate multiple cell death pathways and activate ECS receptors, contributing to tumor regression.

## Discussion

Cannabinoids have gained significant attention in cancer research owing to their potential antitumor effects [24]. Among them, Δ^9^-THC and CBD have been extensively studied for their anti-cancer properties. Δ^9^-THC interacts with the ECS, specifically binding to CB1 receptors, which triggers cancer cell apoptosis and autophagy in gliomas and other cancers [25]. Similarly, cannabidiol inhibits cancer cell proliferation through CB2 receptor activation, inducing cell death in breast and lung cancers [26]. Although some studies suggest that CBC might modulate inflammation and neurological functions through ECS receptors, such as TRPV1 and CB2, a significant gap in the understanding of its direct anti-cancer mechanisms remains [27]. Unlike Δ^9^-THC and CBD, for which pathways that induce cancer cell death are well-documented, the precise biological effects of CBC on tumor progression remain largely unknown. This highlights the need for further research to elucidate the specific functional roles of CBC in cancer therapies.

Our mRNA-seq analysis revealed that CBC treatment upregulated the expression of genes related to apoptosis, ferroptosis, and oxidative stress, while downregulating those involved in cell cycle progression and DNA replication. Previous studies have demonstrated that cannabinoids activate specific signaling pathways that lead to programmed cell death. For example, cannabidiol alters the balance between BAX and Bcl-2 proteins, intrinsically regulating the apoptotic pathway [28]. Moreover, CBD treatment upregulates the expression of cell death-related proteins, such as p53, PARP, RIP1, RIP3, Atg12, and Beclin, suggesting that CBD regulates multiple forms of cell death. Additionally, CBD induces the expression of E-cadherin, peroxisome proliferator-activated receptors-γ (PPARγ), clathrin, β-adaptin, and Tsg101, which are associated with cellular differentiation and vesicle formation [29]. Metallothionein family genes are also regulated during CBD-induced apoptotic cell death, indicating their specific role in this process [30]. CBD increases mitochondrial membrane permeability, leading to cytochrome c release and the activation of extrinsic apoptotic pathways [31]. Similarly, Δ^9^-THC binds to CB1 and CB2 receptors in cancer cells, triggering apoptosis through a cascade of intracellular events. Δ^9^-THC also regulates vesicle formation and cell death [32]. In our study, CBC treatment induced cell death in pancreatic cancer cells, disrupting the cell cycle by increasing the sub-G1 phase and upregulating expression of the tumor suppressor p53. CBC also increased the levels of cleaved forms of PARP-1, caspase-3, and caspase-9, while downregulating the expression of cyclins, including cyclin D3, cyclin E, and cyclin B1. Whereas plant extracts such as curcumin can inhibit cell cycle progression and reduce inflammation and EGCG induces apoptosis and inhibits angiogenesis [33], cannabinoids including CBD and Δ^9^-THC share common pathways that induce cell death through mechanisms such as caspase activation, mitochondrial membrane modulation, and increased oxidative stress. Therefore, the apoptosis-inducing effects of CBC in pancreatic cancer cells seem to reflect a common molecular trait shared by cannabinoids, involving the disruption of key survival pathways and the activation of intrinsic and extrinsic apoptotic signals.

Ferroptosis is a novel form of regulated cell death characterized by iron dependency and the accumulation of lipid peroxides during cell death [34]. Among ferroptosis-related genes, *HMOX1* contributes to ROS production and increases intracellular ferric iron levels by inhibiting GPX4, a crucial enzyme involved in preventing ferroptosis [35]. Several anti-cancer drugs induce ferroptosis through different mechanisms. Erastin, one of the earliest discovered ferroptosis inducers, is a small molecule that inhibits the cysteine/glutamate antiporter system [36]. By blocking cysteine uptake, erastin depletes intracellular glutathione, an antioxidant critical for cellular defense [37]. Another potent ferroptosis inducer, RSL3, operates through the direct inhibition of GPX4, which impairs the ability of the cell to neutralize lipid peroxides, thereby promoting ferroptosis [38]. Beyond these targeted inducers, broad-spectrum drugs such as sorafenib, a multi-kinase inhibitor used to treat hepatocellular carcinoma, also trigger ferroptosis by inhibiting the cystine/glutamate antiporter and reducing glutathione levels [39]. In the present study, CBC regulated the expression of ferroptosis-related genes, including *HMOX1*, while simultaneously inducing ROS production and lipid peroxidation. The CBC-mediated induction of ferroptosis was reversed after co-treatment with ferrostatin-1, a known ferroptosis inhibitor, suggesting that CBC might function as a novel ferroptosis inducer. Moreover, increased HMOX1 expression was observed in CBC-treated xenografts based on IHC analysis, further indicating that CBC tightly regulates HMOX1, both in vitro and in vivo. Notably, the ferroptotic mechanisms associated with other cannabinoids, such as CBD and Δ^9^-THC, have not been fully elucidated. Although this regulation of ferroptosis might represent a unique mechanism associated with CBC, we cannot exclude the possibility that it reflects a broader pharmacological trait common to cannabinoids. Additionally, our results showed that co-treatment with Z-VAD-FMK and ferrostatin-1 restores CBC-induced cell death, suggesting that CBC simultaneously induces apoptosis and ferroptosis, thereby enhancing its anti-cancer efficacy. Iberverin is a natural compound extracted from *Brassica oleracea* var. *capitata*, which induces ferroptosis in hepatocellular carcinoma cells by downregulating SLC7A11 and promoting GPX4 degradation [40]. In contrast, triptolide, another natural compound, inhibits SLC7A11 and GPX4 activity, leading to ferroptosis-induced cardiotoxicity [41]. Isoliquiritigenin, a compound extracted from licorice root, induces ferroptosis in gallbladder cancer cells by promoting *HMOX1* expression [42]. Similarly, the curcumin synthetic analog EF24 has been reported to induce ferroptosis in osteosarcoma by upregulating *HMOX1* and increasing MDA and ROS levels [43]. The above evidence suggests that ferroptosis may be triggered either via the *GPX4*-dependent pathway or via an alternative mechanism involving *HMOX1*. The present study demonstrates that CBC treatment induces ferroptosis by upregulation of *HMOX1*, but does not exclude the possibility of ferroptosis induction via *GPX4*.

The pharmacological activity of cannabinoids is primarily mediated by the ECS, a signaling network responsible for maintaining homeostasis in the body [44]. The ECS consists of two main receptors, CB1 and CB2, which are distributed across various tissues and organs [45]. CB1 receptors are predominantly found in the central nervous system, including the brain and spinal cord. In contrast, CB2 receptors are more abundant in peripheral tissues, particularly immune cells, where they play a role in modulating inflammation and immune responses [46]. Besides interacting with ECS receptors, cannabinoids affect also non-ECS receptor targets, such as the serotonin receptor 5-HT1A, the non-selective TRPV1 cation channel with preference for calcium ions [47], and PPARs, explaining their broad physiological effects [48]. Owing to structural differences, Δ^9^-THC and CBD exhibit distinct interactions with cannabinoid receptors. Δ^9^-THC is known to act as a partial agonist of CB1 and CB2 receptors [49]. Instead, CBD interacts with multiple receptors, including CB1, CB2, GPR55, and TRPV1 [50], but acts as an antagonist of the CB1 receptor [49]. This difference in CB1 receptor activation explains why THC, but not CBD, produces psychoactive effects such as euphoria, altered cognition, and pain relief [51]. Our experiments using Annexin V/PI staining and FACS analysis revealed that CBC-induced cell death was significantly affected when CB2 and TRPV1 were silenced using siRNA, indicating that CBC might exert its anti-cancer effects through these two receptors. Interestingly, after CB2 and TRPV1 inhibitor treatment, cleavage of the apoptosis markers caspase-9 and caspase-3 was diminished, whereas *HMOX1*, a key gene associated with ferroptosis, was unaffected. This suggests that the CB2- and TRPV1-mediated apoptotic effects of CBC might have a stronger influence on apoptosis than ferroptosis. Whereas CBC engages both apoptotic and ferroptotic pathways, the CB2 and TRPV1 receptors appear to predominantly regulate the apoptotic aspect of cell death, implying a more nuanced role for CBC in cancer therapy that could vary depending on the targeted cell death mechanism.

**Fig. 8:**
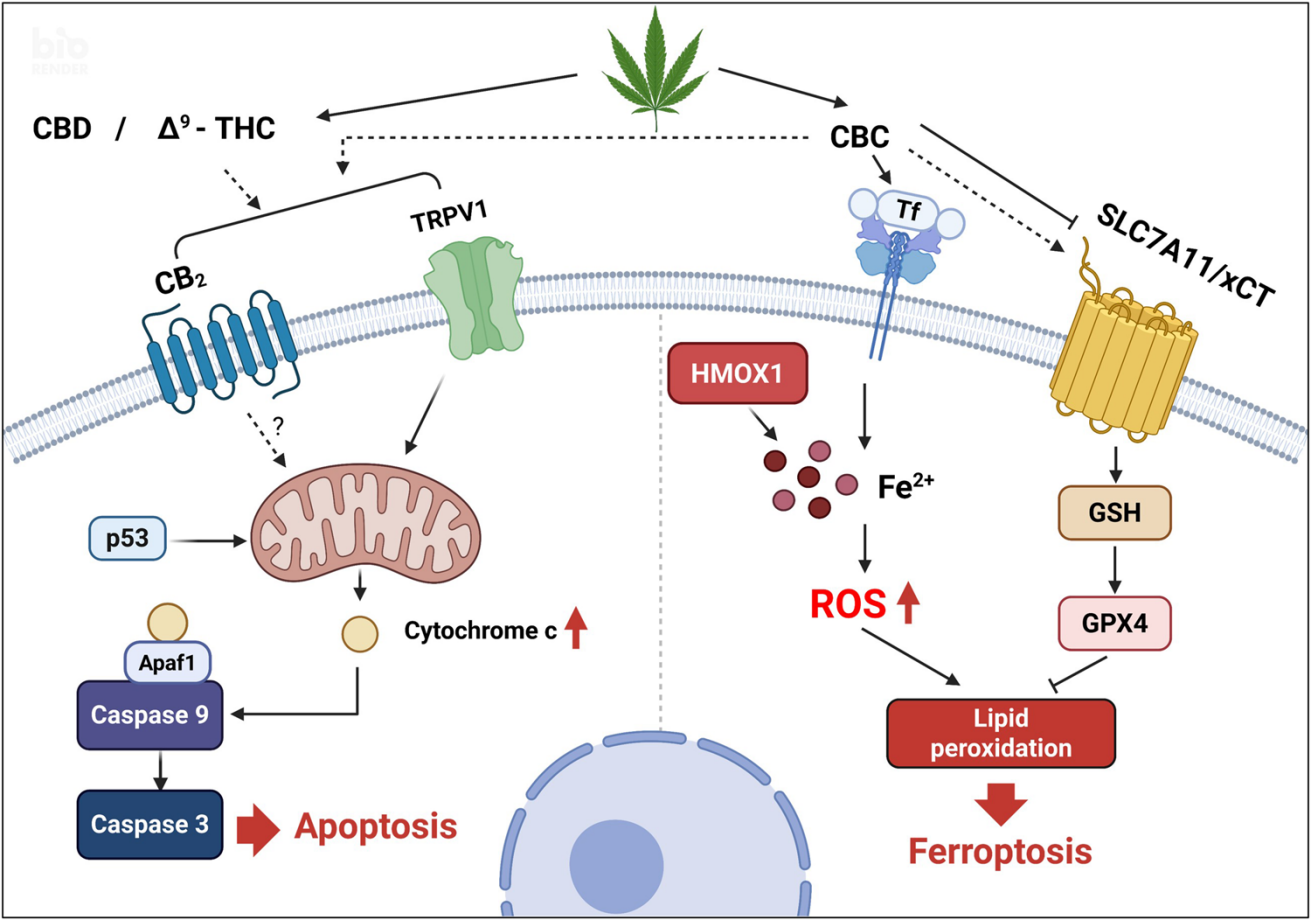
Schematic diagram showing the multiple cell death pathways affected by cannabichromene (CBC) treatment. CBC regulates cell death pathways associated with apoptosis, ferroptosis, and the activation of cannabinoid receptors in an integrative manner.

Our current study demonstrates that CBC modulates multiple forms of cell death by regulating the expression of proteins involved in both apoptotic and ferroptotic pathways. Although CBC-induced apoptosis was dependent on TRPV1 and CB2 receptors, the ferroptotic pathway appeared to be independent of these receptors (Fig. 8). Accordingly, we propose that CBC exerts its pharmacological effects through the integrative modulation of multiple cell death pathways, which could offer therapeutic benefits for pancreatic cancer treatment. These results enhance our understanding of how CBC induces diverse cell death mechanisms via ECS receptors, not only in pancreatic cancer but also in other cancer models. This study provides a promising foundation for the development of cannabinoid-based anti-cancer drugs, offering a new strategy for targeting various types of cancer through the modulation of apoptosis and ferroptosis.

## Materials and methods

### Cell culture and reagents

MIA PaCa-2 and PANC-1 human pancreatic cancer cells were purchased from the non-profit Korean Cell Line Bank (Seoul, Korea). Cells were maintained and grown in Dulbecco’s modified Eagle’s medium (DMEM; Welgene, Seoul, Korea) supplemented with 10% fetal bovine serum (Merck Millipore, MA, USA) and 1% penicillin–streptomycin (Welgene). Cells were cultured at 37 °C in a 5% CO_2_ incubator (Thermo Fisher Scientific, Waltham, MA, USA). The cellular morphology was observed using a phase-contrast microscope (Ts100; Nikon, Tokyo, Japan). CBC, CBD, and Δ^9^-THC were purchased from Cayman Chemical (Ann Arbor, Michigan, USA). Additionally, CBC was extracted and purified in-house via high-performance liquid chromatography using hemp plants cultivated at the Chuncheon Bio Foundation. CBC was dissolved in acetonitrile, and aliquots were stored at −20 °C. We added 35 µM and 30 µM CBC to MIA PaCa-2 and PANC-1 cells, respectively. CB2 and TRPV1 antagonists were purchased from Abcam (Cambridge, UK) and added at a concentration of 10 µM.

### MTT assay

Cell viability was measured using MTT (3-(4,5-dimethylthiazol-2-yl)-2,5-diphenyltetrazolium bromide) assays. The MTT reagent was diluted in phosphate-buffered saline (PBS). Approximately, 7 × 10^3^ cells/well were seeded in 96-well plates, and the following day, each well was treated with CBC in pentaplicate and incubated for the indicated periods. Then, 200 μL of 1 × MTT solution was added and the plates and incubated at 37 °C for 3 h. After incubation, the MTT solution was discarded, and the intracellular purple insoluble formazan was solubilized by adding 100 μL of dimethylsulfoxide. After shaking the plate, absorbance was measured at 570 and 690 nm using a microplate reader (Bio-Rad, Hercules, CA, USA). The half-maximal inhibitory concentration (IC_50_) for cell viability was calculated using GraphPad Prism 8.0.

### Flow cytometric analysis

Pancreatic cancer cells (6 × 10^5^ cells) were seeded in 60-mm dishes and cultured for 24 h at 37 °C in a 5% CO_2_ incubator. The cells were treated or not treated with CBC for 12, 24, or 48 h. After treatment, the cells were harvested, washed twice with PBS, and fixed in 75% ethanol for 24 h. Following fixation, cells were washed three times with PBS to remove residual ethanol and incubated at 37 °C with RNase A (20 μg/mL). The cells were stained with propidium iodide (50 μg/mL) for 30 min. Cell cycle distribution was analyzed using flow cytometry (FACSymphony, Bergen County, NJ, USA) according to the manufacturer’s protocol.

### Annexin V/propidium iodide (PI) staining

Approximately, 2 × 10^5^ cells/well were seeded in 6-well plates and cultured for 24 h at 37 °C in a 5% CO_2_ incubator. Next, the cells were treated with CBC for 24 h. Apoptosis in pancreatic cancer cells was assessed using a double-staining method comprising Annexin V-FITC and PI (BioVision, Milpitas, CA, USA). The cells were incubated with 50 μL of Annexin V-FITC for 10 min in the dark at room temperature, and PI was added. The apoptotic cells were analyzed using a flow cytometer (FACSymphony, Bergen County, NJ, USA) and visualized using a confocal microscope (Nikon, Minato, Tokyo, Japan).

### Western blot analysis

Approximately, 7 × 10^6^ cells were seeded in 100-mm dishes and cultured for 24 h at 37 °C in a 5% CO_2_ incubator. Next, the cells were treated or not treated with CBC for 12, 24, and 48 h. Proteins were extracted using radioimmunoprecipitation assay lysis buffer (25 mM Tris-HCl [pH 7.6], 150 mM NaCl, 1% NP-40, 1% sodium deoxycholate, and 0.1% sodium dodecyl sulfate) containing protease inhibitors. The total protein content was determined using the Bradford reagent (Bio-Rad, Hercules, CA, USA), and 30 µg of protein was separated via sodium dodecyl sulfate-gel electrophoresis and transferred onto a polyvinylidene fluoride (PVDF) membrane. The PVDF membrane was blocked with 5% skim milk for 30 min at room temperature and incubated with the primary antibody overnight at 4 °C. After washing, the membranes were incubated with HRP-conjugated goat anti-mouse IgG or HRP-conjugated goat anti-rabbit IgG secondary antibodies at room temperature for 1 h. Finally, proteins were detected using an ECL protein detection kit (GE Healthcare, Chicago, IL, USA). Primary antibodies against PARP-1 (Cat. # 9542 RRID:AB_2160739), caspase-9 (Cat. # 9502 RRID:AB_2068621), and caspase-3 (Cat. # 9665 RRID:AB_2069872) were purchased from Cell Signaling Technology (Danvers, MA, USA). Antibodies against p53 (Cat. # sc-126, RRID:AB_628082), cyclin E (Cat. # sc-247, RRID:AB_627357), cyclin D3 (Cat. # sc-6283, RRID:AB_627355), cyclin B1 (Cat. # sc-245, RRID:AB_627338), and β-actin (Cat. # sc-47778, RRID:AB_626632) were purchased from Santa Cruz Biotechnology (Dallas, TX, USA). Anti-HMOX1 (Cat. # ab68477, RRID:AB_11156457), anti-GPX4 (Cat. # ab125066, RRID:AB_10973901), anti-Ki67 (Cat. # ab16667, RRID:AB_302459), and anti-cannabinoid receptor 2 (Cat. # ab3561, RRID:AB_303908) antibodies was obtained from Abcam. Finally, the antibody against TRPV1 (Cat. # PA1-748, RRID:AB_2209010) was purchased from Invitrogen (Thermo Fisher Scientific).

### Immunostaining

Cells were cultured on glass coverslips, washed with PBS, and fixed with a 4% paraformaldehyde before permeabilization with 0.1% Triton X-100 in PBS for 5 min. After blocking for 1 h with 5% skim milk in PBS, the slides were incubated with the primary antibody (1:100) at room temperature for 1 h. The primary antibody was prepared using 5% skim milk. After washing with PBS, the cells were incubated with an Alexa 488-conjugated goat anti-mouse IgG secondary antibody (1:200; Abcam, Boston, MA, USA), which was diluted 1:500 in 5% skim milk. Cells were counterstained with DAPI.

### Quantitative real-time polymerase chain reaction (qRT-PCR) analysis

Total RNA was isolated using TRIzol reagent (Ambion, North Greenbush, NJ, USA). The quality and quantity of RNA were assessed using a spectrophotometer (BMG Labtech, Ortenberg, Germany). Complementary DNA (cDNA) was synthesized in a 20 μL reaction mixture containing 1 μg RNA, oligo dT, 5 × buffer, 10 mM dNTP, and M-MLV reverse transcriptase. qRT-PCR analysis was performed using SYBR Green qPCR PreMIX (Enzynomics, Daejeon, Korea) according to the manufacturer’s instructions. The total reaction volume was 20 μL, and it contained cDNA, the qPCR mix, and primers. The PCR reaction conditions were as follows: initial denaturation at 95 °C for 10 min, followed by 40 cycles of 95 °C for 15 s and 60°C for 1 min. Melting curve analysis was conducted from 60 °C to 95 °C, with readings taken every second. The 2^-ΔΔCT comparative method was used for relative gene expression quantification, with CT values normalized to those of the *β-actin* housekeeping gene. Primers were purchased from Genotech, Inc. (Daejeon, Korea). The target sequences were amplified using the following primers:

**Table:** 

HMOX1	F	5′-AGGTCATCCCCTACACACCA-3′
	R	5′-TCTGGGCAATCTTTTTGAGC-3′
CHAC1	F	5′-TGGTGACGCTCCTTGAAGAT-3′
	R	5′-CCAGACGCAGCAAGTATTCA-3′
SLC3A2	F	5′-TCTTGATTGCGGGGACTAAC-3′
	R	5′-GCCTTGCCTGAGACAAACTC-3′
SLC7A11	F	5′-CCACTGTTCATCCCAGCTTT-3′
	R	5′-CCTGGGTTTCTTGTCCCATA-3′
FTH1	F	5′-GACCCCCATTTGTGTGACTT-3′
	R	5′-CAGGGTGTGCTTGTCAAAGA-3′

### Library preparation and sequencing

Libraries were prepared from total RNA using the NEBNext Ultra II Directional RNA-Seq Kit (New England BioLabs Inc., UK), and mRNA was isolated using a Poly(A) RNA Selection Kit (Lexogen, Inc., Austria). Isolated mRNA was subsequently used for cDNA synthesis and sharing following the manufacturer’s instructions. Indexing was conducted using Illumina Indexes 112. The enrichment step was performed using PCR. The libraries were assessed using a TapeStation HS D1000 Screen Tape (Agilent Technologies, Amstelveen, The Netherlands) to evaluate the mean fragment size. Quantification was performed using a library quantification kit in conjunction with a StepOne Real-Time PCR System (Life Technologies). High-throughput sequencing was performed as paired-end 100 sequencing using NovaSeq 6000 (Illumina, Inc., USA).

### RNA-seq data analysis

Raw sequencing data quality control was performed using Fast QC (Simon, 2010). Adapter and low-quality reads (<Q20) were removed using cutadapt software (version 2.7). The trimmed reads were aligned to the *Homo sapiens* reference genome (GRCh38) using STAR software with FTH1defaFult parameters [52]. After alignment, the numbers of reads mapped to gene features (GTF file of GRCh38.89) were counted using HTSeq [53]. Subsequently, read counts for the samples in each condition were normalized using the trimmed mean of M-values normalization in the edgeR package [54]. Differentially expressed genes (DEGs) were identified by log_2_-fold changes >1 when comparing the CBC-treated sample with its respective vehicle control in each cell line; for example, MIA PaCa-2 (CBC) vs. MIA PaCa-2 (control) and PANC-1 (CBC) vs. PANC-1 (control). Gene set enrichment analysis of DEGs was performed using DAVID software [55]. Gene Ontology (GO) biological processes and DEG-associated enriched pathways were identified as those with *p*-values < 0.05. Raw RNA-seq data were deposited in the Gene Expression Omnibus (GEO) database under accession ID GSE291851.

### Transfection

siRNAs were purchased from Bioneer, and non-target siRNA (NT siRNA) was used as a negative control. For siRNA transfection, 2 × 10^5^ pancreatic cancer cells per well were seeded in 60 or 100 mm culture dishes in serum-free medium. After 4 h, CNR2, TRPV1, and HMOX1 along with NT siRNA were transfected into the cells using Lipofectamine 2000 transfection reagent (Invitrogen), following the manufacturer’s instructions.

**Table:** 

Gene	siRNA #	Sequence (5′-3′)	
CNR2	1269-1	CAGUUCACUCCCUGGAAGA	UCUUCCAGGGAGUGAACUG
	1269-2	GUCAAGAAGGCCUUUGCUU	AAGCAAAGGCCUUCUUGAC
TRPV1	7442-1	GAUUGAAGACGGGAAGAAU	AUUCUUCCCGUCUUCAAUC
	7442-2	GUGUUAGGAGGAGUCUACU	AGUAGACUCCUCCUAACAC
HMOX1	3162-1	CAAAAAGAUUGCCCAGAAA	UUUCUGGGCAAUCUUUUUG
	3162-2	CACAAACCUGAAAAGAUGU	ACAUCUUUUCAGGUUUGUG

### Reactive oxygen species (ROS) analysis

The cells were cultured in 60 mm culture dishes overnight to facilitate adhesion and treated with the reagent for 24 h. Subsequently, diluted 10 mM carboxy-H_2_DCFDA (Invitrogen) was added to the medium, followed by incubation at 37 °C for 30 min. Cells were gently washed twice with warm PBS. ROS levels were determined via flow cytometry using 488 nm excitation and 525 nm emission wavelengths (FACSymphony, Bergen County, NJ, USA).

### Lipid peroxidation

Relative lipid peroxidation levels in the cells were assessed using a lipid peroxidation kit (DoGenBio, Seoul, Korea). Cells were seeded in 60 mm culture dishes 24 h before treatment with CBC and ferroptosis-related reagents. Approximately, 7 × 10^5^ cells were seeded. Cells were harvested after treatment with different reagents for 24 h, and lipid peroxidation was analyzed according to the manufacturer’s instructions.

### GSH assay

The relative GSH/GSSG ratio in the cell lysates was assessed using a Glutathione Assay Kit (Biomax, Guri, Korea) according to the manufacturer’s instructions. The total glutathione content was calculated by measuring the optical density at 412 nm.

### Animal experiment

MIA PaCa-2 pancreatic cancer cells were used to establish a xenograft tumor model. Cells were harvested via trypsinization and resuspended in DMEM. Then, 8 × 10^6^ cells in 50 μL were subcutaneously injected into 6-week-old male nude mice. Eleven mice were randomly assigned to three groups, PBS, CBC, and Fer-1 + CBC (*n* = 3 or 4 per group). CBC (5 mg/kg) was administered via subcutaneous injection every 2 days, starting 22 days after tumor implantation. The mice were euthanized on day 32 to harvest the tumors. Mouse experiments were approved by the Institutional Animal Care and Use Committee of Kangwon National University (KW-240320-2). Mice were housed under specific pathogen-free conditions at the animal facility and handled according to the guidelines by the Institutional Animal Care and Use Committee.

### Hematoxylin and eosin (H&E) staining

Tissue sections were deparaffinized using xylene and then rehydrated using a graded series of ethanol and water. Subsequently, the sections were stained with H&E. The stained slides were scanned at 40× magnification.

### Immunohistochemistry (IHC)

IHC was performed on tumors fixed overnight in 4% formalin. The tissue sections were deparaffinized and rehydrated according to the protocol for H&E staining and then heated at 90 °C in citrate buffer (Diagnostic Biosystems, Pleasanton, CA, USA) for 10 min. Subsequently, sections were incubated with 3% hydrogen peroxide (Walgreens, Deerfield, IL, USA) for 5 min to block endogenous peroxidase activity. The sections were then incubated overnight at 4 °C with anti-Ki67, anti-CB2, anti-TRPV1, anti-p53, anti-cleaved caspase-3, anti-HMOX1, or anti-GPX4 antibodies. The following day, the sections were incubated with a conjugated secondary antibody for 30 min and developed using DAB (Agilent/Dako, Santa Clara, CA, USA), followed by hematoxylin staining. The sections were then dehydrated, and the stained slides were scanned at a 40× magnification.

### Statistical analysis

Statistical analyses were performed using GraphPad Prism 8.0, FlowJo, and ImageJ software. The graphs show the mean ± standard deviation of at least three independent experiments. GO and KEGG pathway analyses were performed using DAVID software. Statistical significance was determined using Student’s *t* tests, with *p*-values ≤ 0.05 indicating statistical significance. (*P < 0.05, **P < 0.01, ***P < 0.001, ****P < 0.0001) Statistical differences were determined by one-way analysis of variance (ANOVA) followed, when significant, by Dunnett’s or Tukey’s multiple comparison post hoc tests. The effects were considered significant at *P* < 0.05.

## Supplementary information

Supplementary Figures

Western blot uncropped image

## Data Availability

The datasets used and/or analyzed during the current study available from the corresponding author on reasonable request.
