# Supplementary material for: Cannabichromene: integrative modulation of apoptosis, ferroptosis, and endocannabinoid signaling in pancreatic cancer therapy
Source: Cell Death Discov. 2025 Aug 11;11:377. doi: 10.1038/s41420-025-02674-8 (PMC12340112; doi:10.1038/s41420-025-02674-8)
Supplement: Supplementary file 1 — Supplementary Figures [file 41420_2025_2674_MOESM1_ESM.docx]

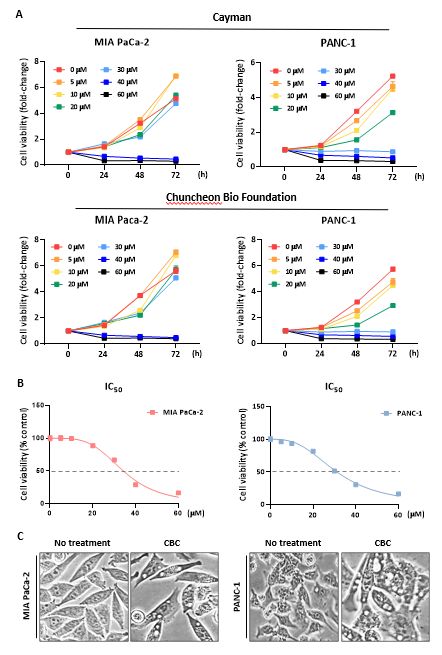


**Supplementary Fig S1. Growth inhibitory effect and morphological changes induced by cannabichromene (CBC) treatment.** (A) An MTT assay was performed to evaluate the growth-inhibitory effects of CBC on MIA PaCa-2 and PANC-1 pancreatic cancer cells. The cell proliferation inhibitory effects of CBC obtained from Cayman Inc. and Chuncheon Bio Foundation were compared (*Upper:* CBC from Cayman Inc.; *Lower:* CBC from Chuncheon Bio Foundation). (B) The IC_50_ values were determined using GraphPad Prism (MIA PaCa-2 = 34.33 μM, PANC-1 = 31.65 μM). (C) Morphological changes and intracellular vesicle formation induced by CBC treatment were also observed.


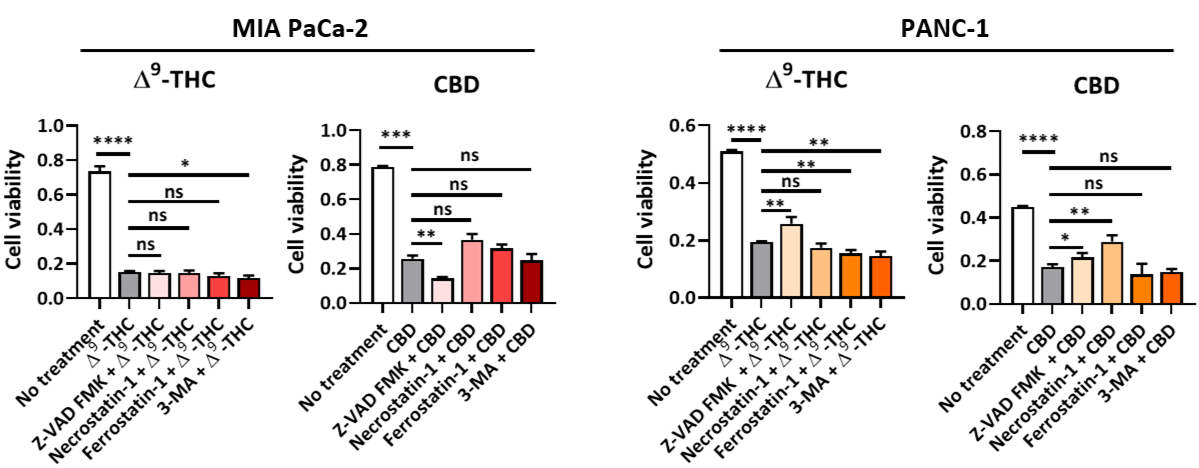


**Supplementary Fig S2. Cell viability changes after combination treatment with several inhibitors and Δ^9^-tetrahydrocannabinol (Δ^9^-THC)** **or cannabidiol (CBD).** Δ^9^-THC treatment reduced cell viability, but combination treatment with several inhibitors did not result in any significant changes. CBD treatment resulted in a significant decrease in cell viability, which was recovered only after combination treatment with Z-VAD-FMK and necrostatin-1. Statistical significance was determined using Student’s t-tests, with p-values ≤ 0.05 indicating statistical significance.


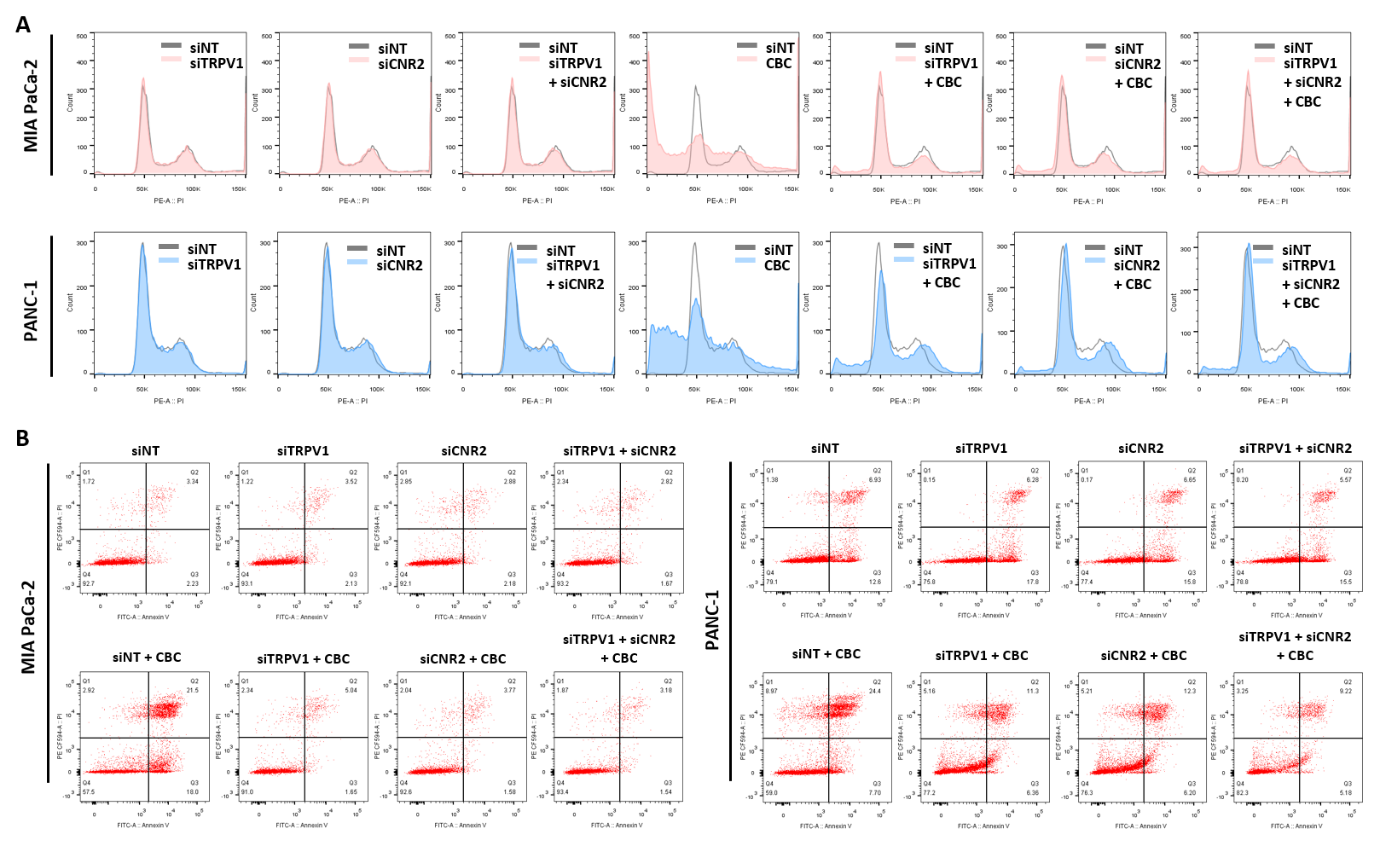


**Supplementary Fig S3. Original data for FACS and AnnexinV/propidium iodide (PI) staining experiment corresponding to the graphs in Figure 6B and C.**


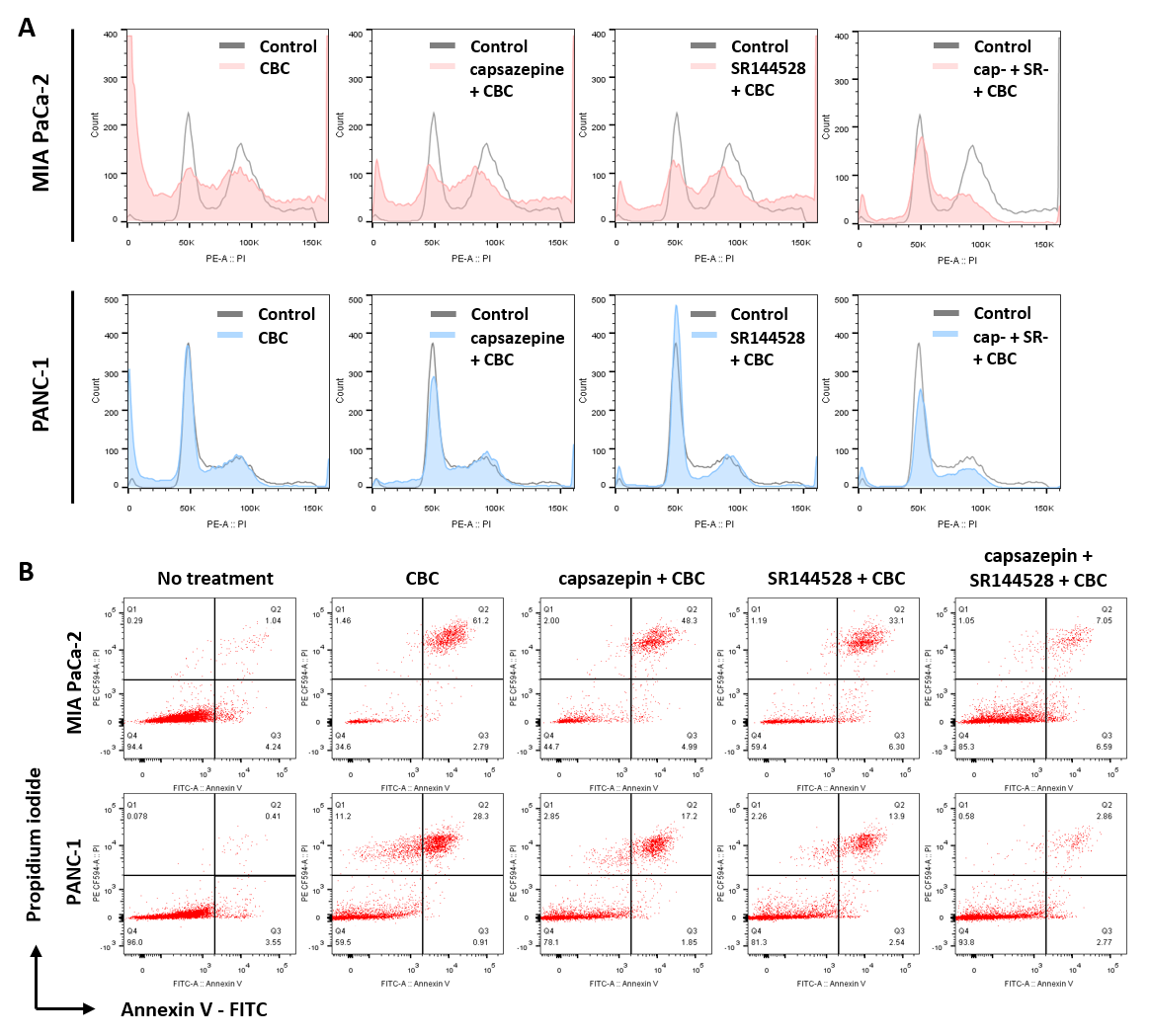


**Supplementary Fig S4. Original data for FACS and AnnexinV/propidium iodide (PI) staining experiment corresponding to the graphs in Figure 6D and E.**


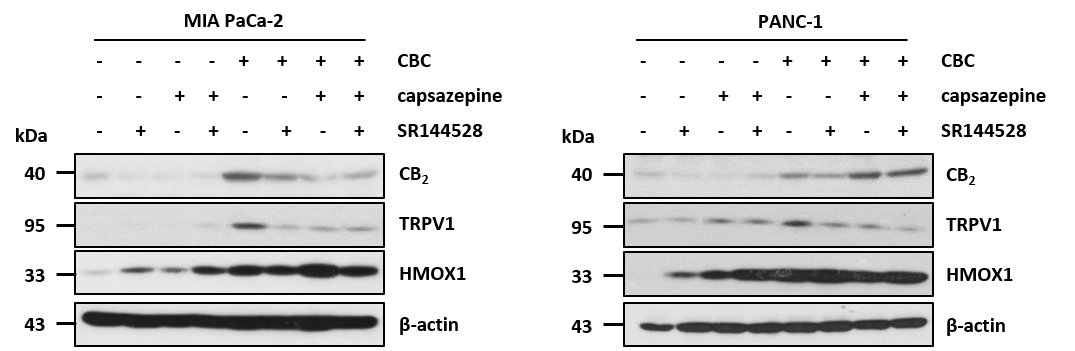


**Supplementary Fig S5. Western blot analysis of HMOX-1 after combination treatment comprising inhibitors of cannabinoid receptors and cannabichromene (CBC).** Western blot analysis showed that HMOX1 protein expression did not change after combination treatment with capsazepine or SR144528 and CBC.
