# Supplementary material for: Cannabichromene: integrative modulation of apoptosis, ferroptosis, and endocannabinoid signaling in pancreatic cancer therapy
Source: Cell Death Discov. 2025 Aug 11;11:377. doi: 10.1038/s41420-025-02674-8 (PMC12340112; doi:10.1038/s41420-025-02674-8)
Supplement: Supplementary file 2 — Western blot uncropped image [file 41420_2025_2674_MOESM2_ESM.pptx]

## Slide 1
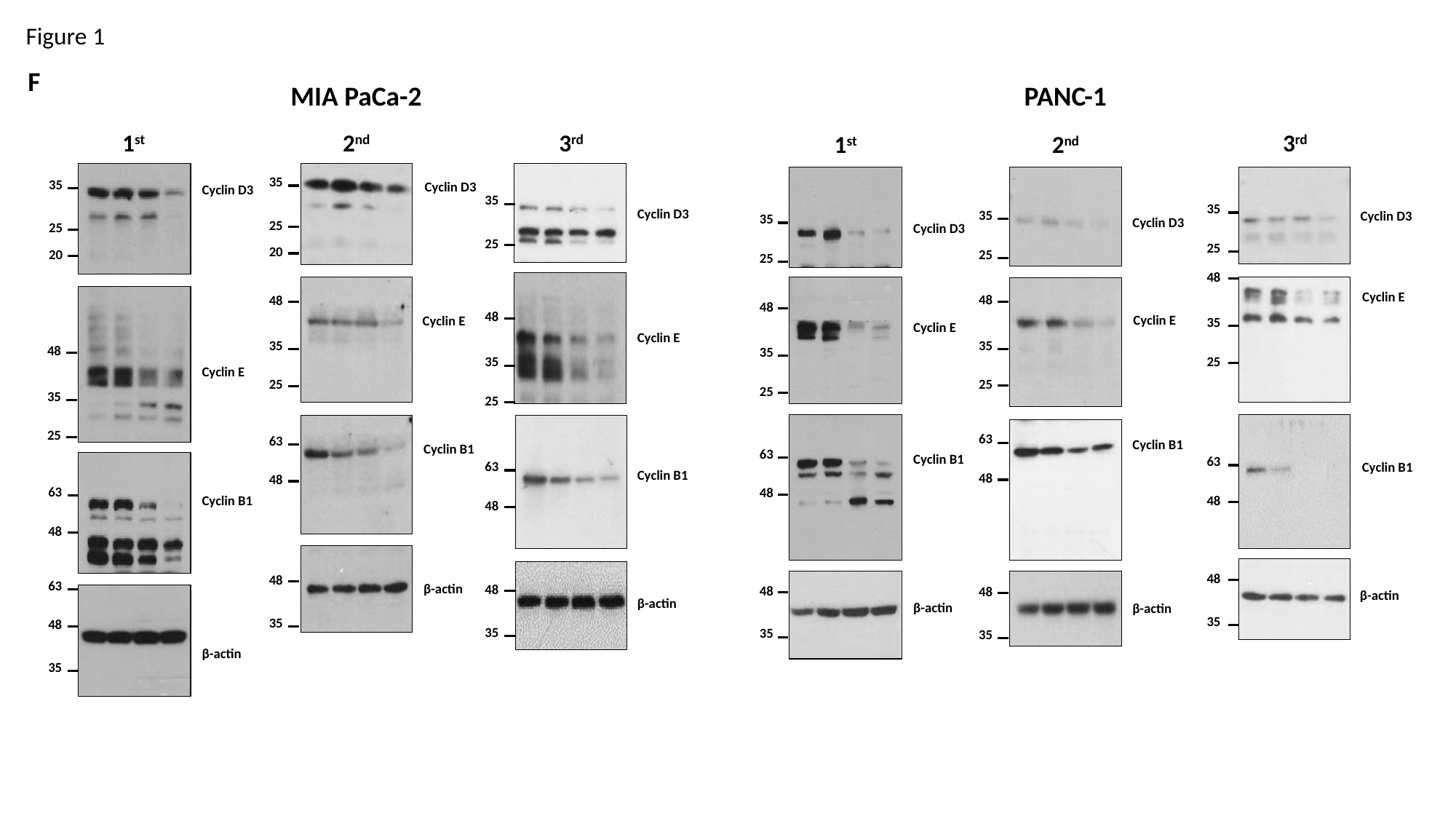

Figure 1
F
MIA PaCa-2
PANC-1
1st
2nd
3rd
3rd
2nd
1st
35
Cyclin D3
25
20
48
Cyclin E
35
25
63
Cyclin B1
48
63
48
β-actin
35
35
Cyclin D3
25
20
48
Cyclin E
35
25
63
Cyclin B1
48
48
β-actin
35
35
Cyclin D3
25
48
Cyclin E
35
25
63
Cyclin B1
48
48
β-actin
35
35
Cyclin D3
25
48
Cyclin E
35
25
63
Cyclin B1
48
48
β-actin
35
35
Cyclin D3
25
48
Cyclin E
35
25
63
Cyclin B1
48
48
β-actin
35
35
Cyclin D3
25
48
Cyclin E
35
25
63
Cyclin B1
48
48
β-actin
35

## Slide 2
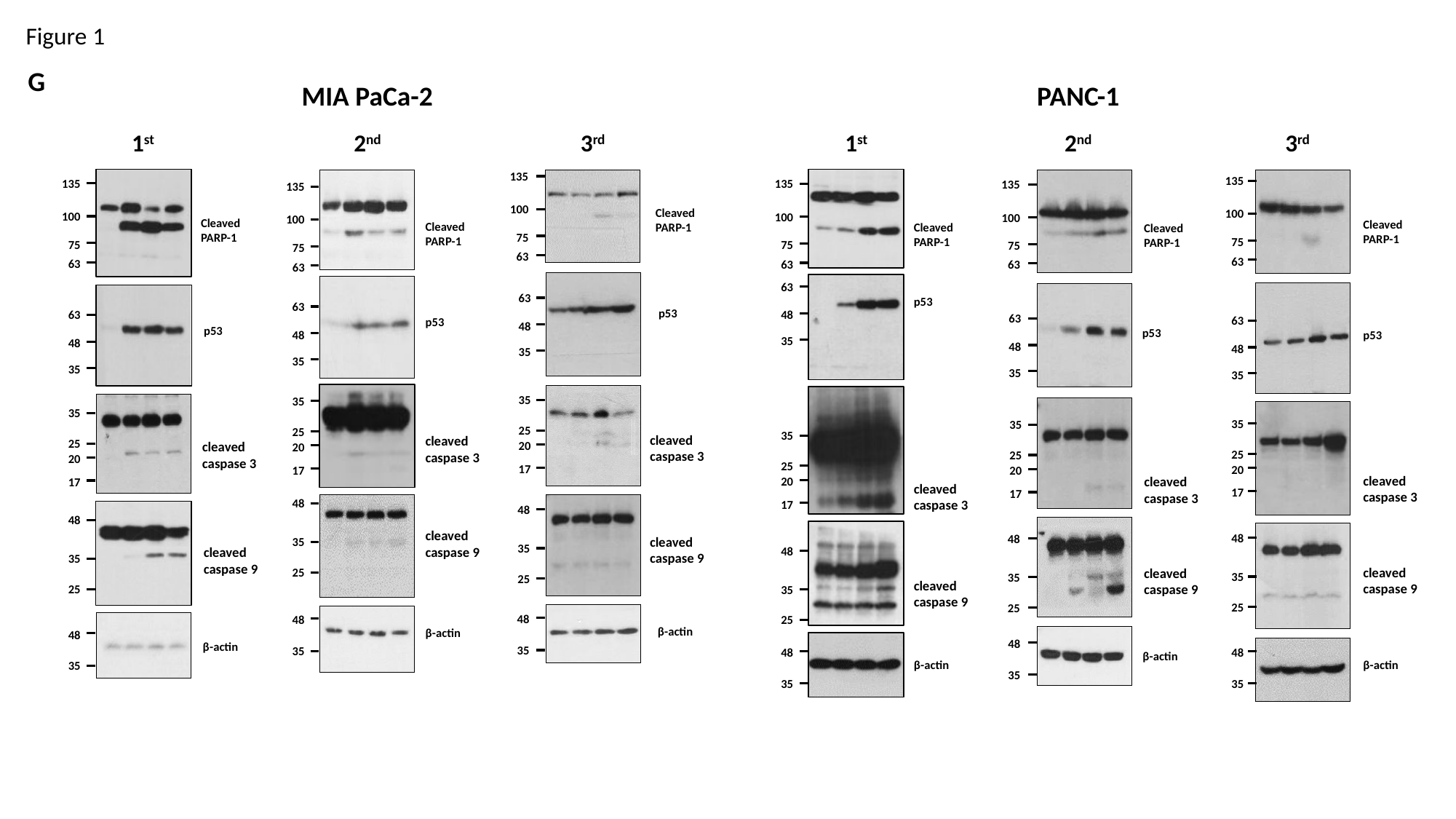

Figure 1
G
MIA PaCa-2
PANC-1
1st
2nd
3rd
1st
2nd
135
100
Cleaved
PARP-1
75
63
63
p53
48
35
35
25
20
cleaved
caspase 3
17
48
cleaved
caspase 9
35
25
48
β-actin
35
3rd
135
100
Cleaved
PARP-1
75
63
63
p53
48
35
35
25
20
cleaved
caspase 3
17
48
cleaved
caspase 9
35
25
48
β-actin
35
135
100
Cleaved
PARP-1
75
63
63
p53
48
35
35
25
cleaved
caspase 3
20
17
48
cleaved
caspase 9
35
25
48
β-actin
35
135
100
Cleaved
PARP-1
75
63
63
p53
48
35
35
25
cleaved
caspase 3
20
17
48
cleaved
caspase 9
35
25
48
β-actin
35
135
100
Cleaved
PARP-1
75
63
63
p53
48
35
35
25
cleaved
caspase 3
20
17
48
cleaved
caspase 9
35
25
48
β-actin
35
135
100
Cleaved
PARP-1
75
63
63
p53
48
35
35
25
20
cleaved
caspase 3
17
48
cleaved
caspase 9
35
25
48
β-actin
35

## Slide 3
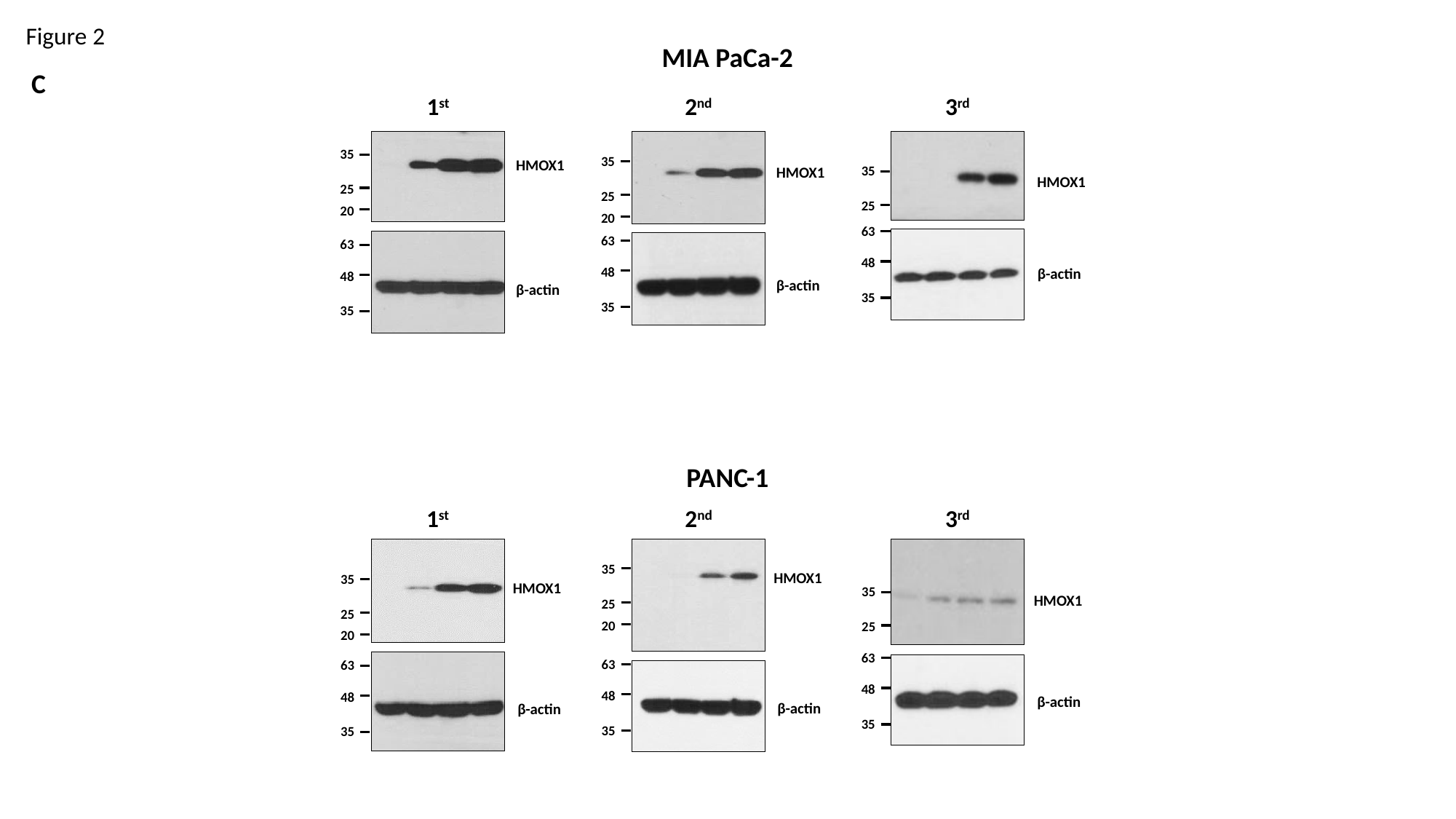

Figure 2
MIA PaCa-2
C
1st
35
HMOX1
25
20
63
48
β-actin
35
2nd
35
HMOX1
25
20
63
48
β-actin
35
3rd
35
HMOX1
25
63
48
β-actin
35
PANC-1
1st
35
HMOX1
25
20
63
48
β-actin
35
2nd
35
HMOX1
25
20
63
48
β-actin
35
3rd
35
HMOX1
25
63
48
β-actin
35

## Slide 4
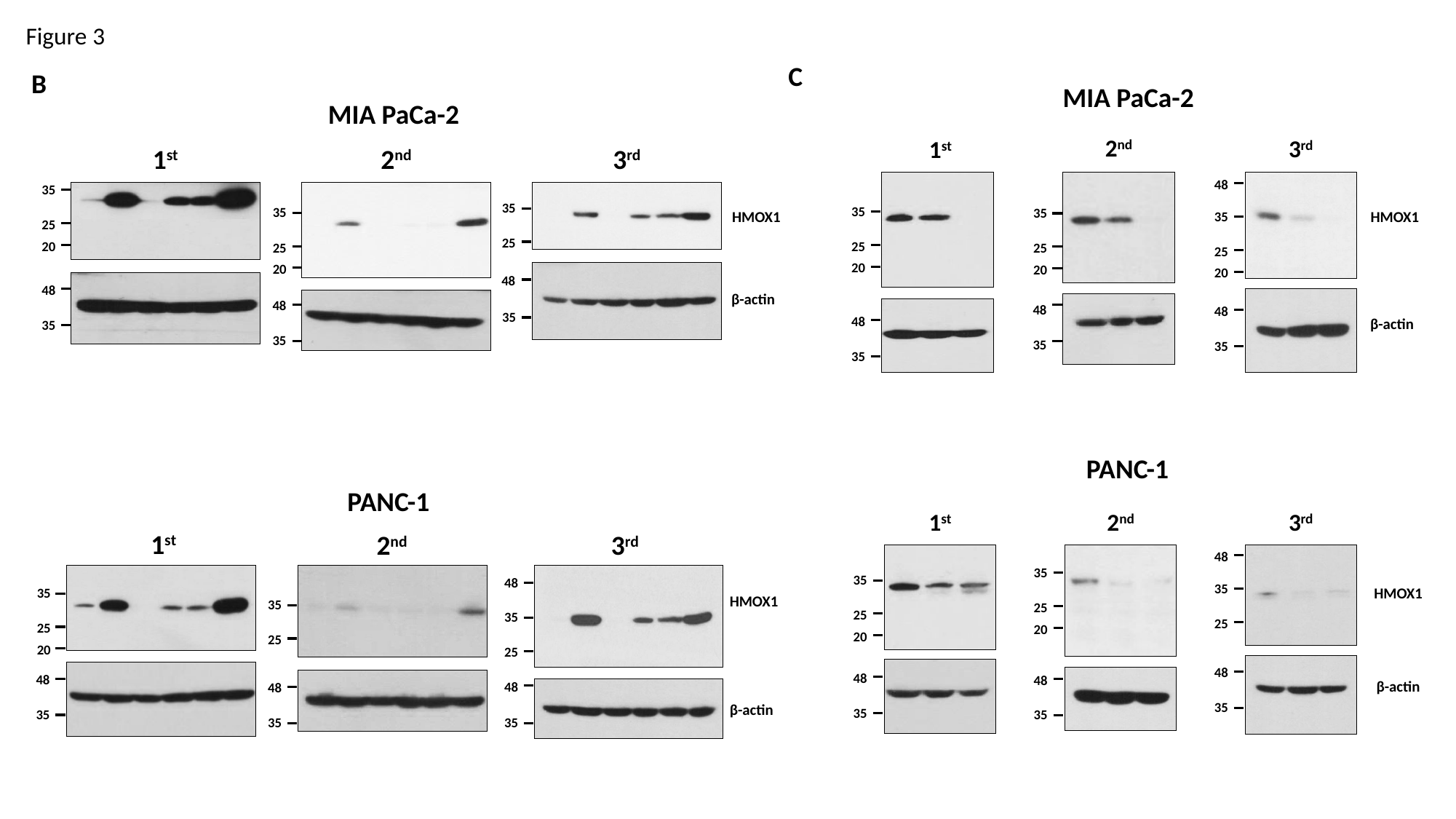

Figure 3
C
B
MIA PaCa-2
MIA PaCa-2
2nd
35
25
20
48
35
3rd
48
HMOX1
35
25
20
48
β-actin
35
1st
35
25
20
48
35
1st
35
25
20
48
35
2nd
35
25
20
48
35
3rd
35
HMOX1
25
48
β-actin
35
PANC-1
PANC-1
1st
35
25
20
48
35
2nd
35
25
20
48
35
3rd
48
35
HMOX1
25
48
β-actin
35
1st
35
25
20
48
35
2nd
35
25
48
35
3rd
48
HMOX1
35
25
48
β-actin
35

## Slide 5
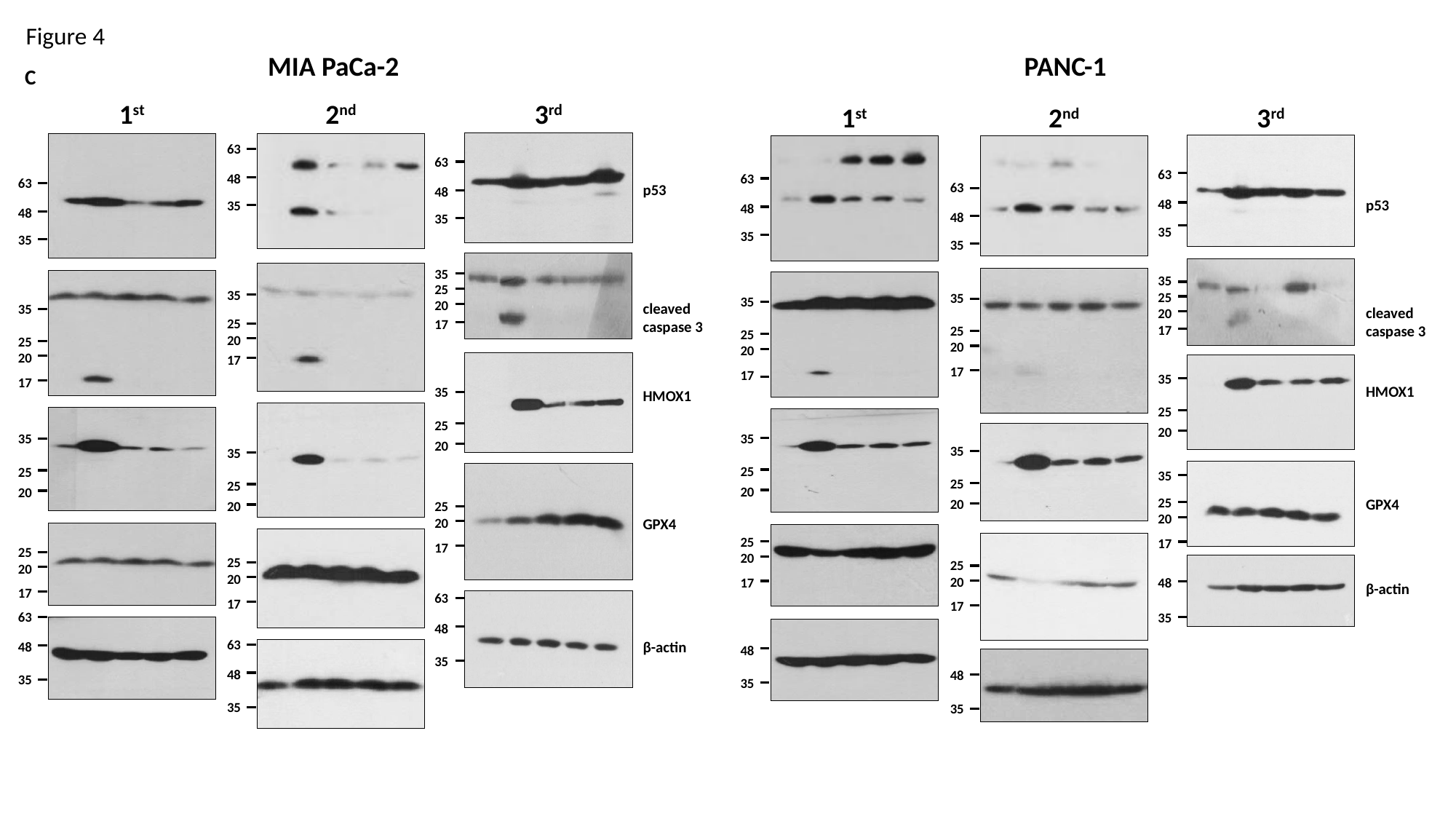

Figure 4
MIA PaCa-2
3rd
63
p53
48
35
35
25
20
cleaved caspase 3
17
35
HMOX1
25
20
25
20
GPX4
17
63
48
β-actin
35
PANC-1
C
1st
63
48
35
35
25
20
17
35
25
20
25
20
17
63
48
35
2nd
63
48
35
35
25
20
17
35
25
20
25
20
17
63
48
35
1st
63
48
35
35
25
20
17
35
25
20
25
20
17
48
35
2nd
63
48
35
35
25
20
17
35
25
20
25
20
17
48
35
3rd
63
p53
48
35
35
25
cleaved caspase 3
20
17
35
HMOX1
25
20
35
25
GPX4
20
17
48
β-actin
35

## Slide 6
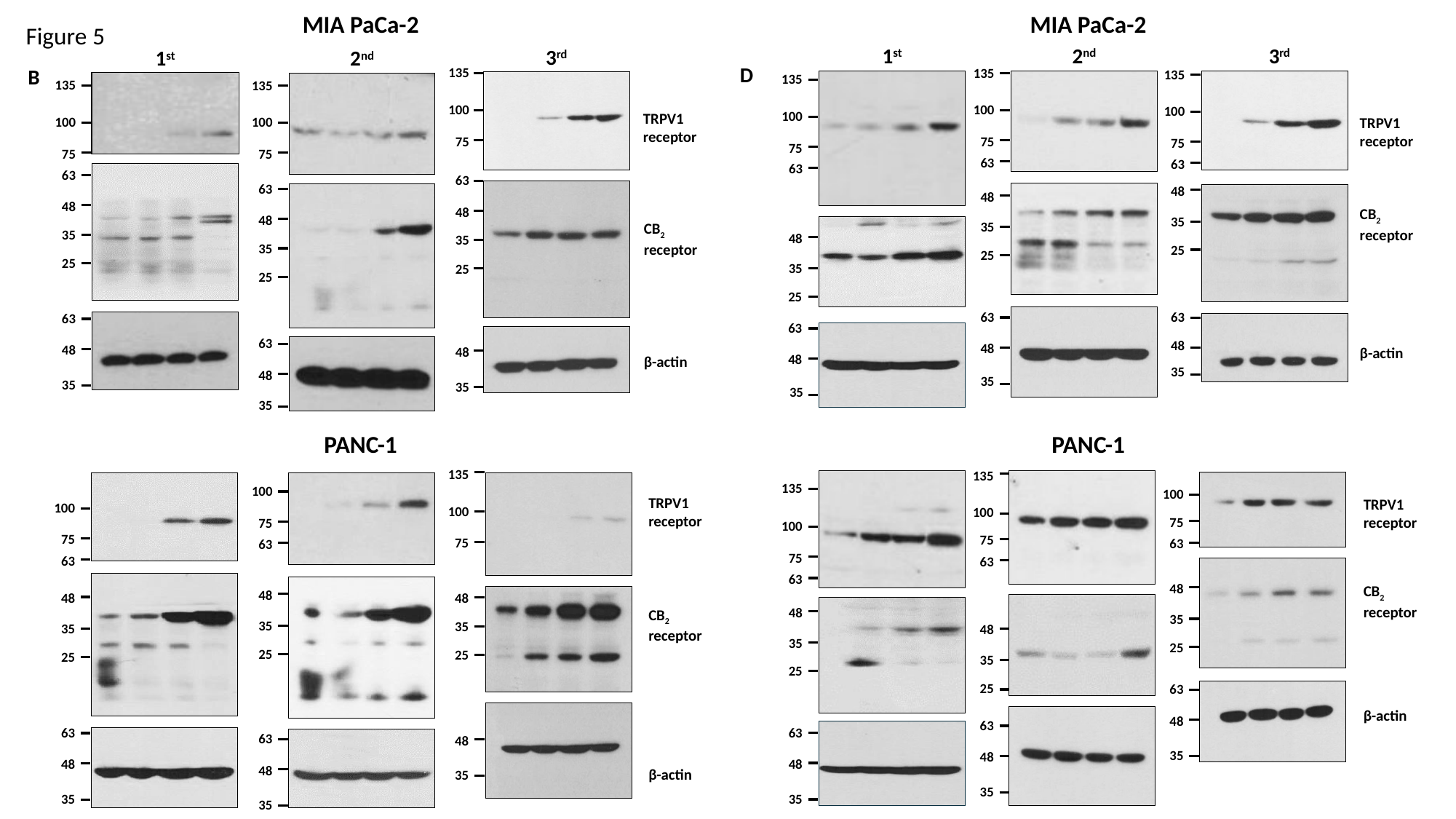

MIA PaCa-2
MIA PaCa-2
Figure 5
1st
135
100
75
63
48
35
25
63
48
35
2nd
3rd
135
100
TRPV1
receptor
75
63
48
CB2
receptor
35
25
63
48
β-actin
35
3rd
135
100
TRPV1 receptor
75
63
48
CB2 receptor
35
25
48
β-actin
35
1st
135
100
75
63
48
35
25
63
48
35
2nd
135
100
75
63
48
35
25
63
48
35
D
B
135
100
75
63
48
35
25
63
48
35
PANC-1
PANC-1
135
TRPV1 receptor
100
75
48
CB2 receptor
35
25
48
β-actin
35
135
100
75
63
48
35
25
63
48
35
135
100
75
63
48
35
25
63
48
35
100
TRPV1
receptor
75
63
48
CB2
receptor
35
25
63
β-actin
48
35
100
75
63
48
35
25
63
48
35
100
75
63
48
35
25
63
48
35

## Slide 7
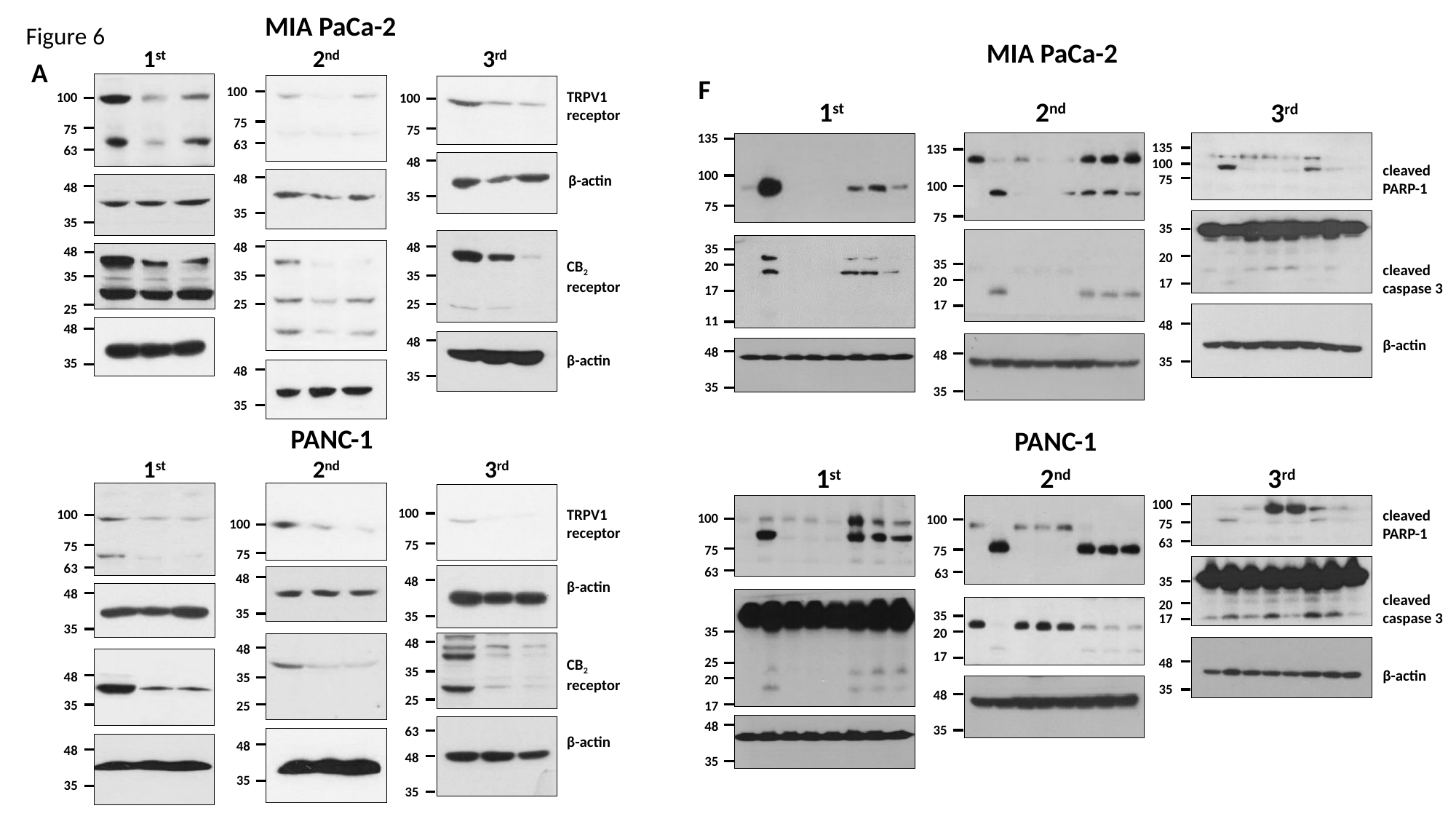

MIA PaCa-2
Figure 6
MIA PaCa-2
1st
100
75
63
48
35
48
35
25
48
35
2nd
100
75
63
48
35
48
35
25
48
35
3rd
TRPV1
receptor
100
75
48
β-actin
35
48
CB2
receptor
35
25
48
β-actin
35
A
F
1st
135
100
75
35
20
17
11
48
35
1st
100
75
63
35
25
20
17
48
35
2nd
135
100
75
35
20
17
48
35
PANC-1
2nd
100
75
63
35
20
17
48
35
3rd
135
100
cleaved PARP-1
75
35
20
cleaved caspase 3
17
48
β-actin
35
3rd
100
cleaved PARP-1
75
63
35
cleaved caspase 3
20
17
48
β-actin
35
PANC-1
1st
100
75
63
48
35
48
35
48
35
2nd
100
75
48
35
48
35
25
48
35
3rd
100
TRPV1
receptor
75
48
β-actin
35
48
CB2
receptor
35
25
63
β-actin
48
35
